# Matrix degradation enhances stress relaxation, regulating cell adhesion and spreading

**DOI:** 10.1073/pnas.2416771122

**Published:** 2025-03-25

**Authors:** Badri Narayanan Narasimhan, Stephanie I. Fraley

**Affiliations:** ^a^Department of Bioengineering, University of California, San Diego, CA 92093

**Keywords:** extracellular matrix, degradability, matrix metalloproteinase, stress relaxation, collagen

## Abstract

In native extracellular matrices (ECM), cells utilize matrix metalloproteinases (MMPs) to degrade and remodel their microenvironment. Accordingly, synthetic matrices have been engineered to permit MMP-mediated cleavage, facilitating cell spreading, migration, and interactions. However, the interplay between matrix degradability and mechanical properties remains underexplored. We hypothesized that MMP activity induces immediate mechanical alterations in the ECM, which are subsequently detected by cells. We observed that both fibrillar collagen and synthetic degradable matrices exhibit enhanced stress relaxation following MMP exposure. Cells responded to these variations in relaxation by modulating their spreading and focal adhesions. Furthermore, we demonstrated that stress relaxation and cell spreading can be precisely controlled through the rational design of matrix degradability. These findings establish a fundamental link between matrix degradability and stress relaxation, with potential implications for a broad spectrum of biological applications.

## Significance

Cells can sense when the extracellular matrix has been exposed to matrix metalloproteinases (MMPs), even if the exposure is slight and does not alter matrix stiffness. We show that cells sense MMP-mediated degradation through its enhancement of stress relaxation in both fibrillar collagen and synthetic degradable matrices. By tuning the degradability of these systems, we demonstrate that we can control degradation-mediated stress relaxation and thereby regulate cellular mechanosensing. Previous efforts to engineer stress-relaxing biomaterials have predominantly used systems with ionic crosslinking. Separately, degradability has been tuned to engineer cellular confinement. Our study establishes a fundamental link between matrix degradability and stress relaxation and demonstrates that stress relaxation can be tuned through the rational design of matrix degradability.

Native extracellular matrix (ECM) is a complex assembly of proteins that imparts essential mechanical and biochemical properties to tissues. Advancements in synthetic biomaterials have enabled precise modulation of ECM mechanical features, enhancing our understanding of cellular mechanotransduction. Variations in crosslinking mechanisms have allowed systematic adjustment of the elastic modulus, elucidating the influence of stiffness on cell differentiation, adhesion, proliferation, and gene expression (1–3). Similarly, tuning viscoelasticity through distinct crosslinking strategies or with interpenetrating polymer networks has revealed impacts on cell behavior, including adhesion, alignment, migration, and ECM remodeling (4–6). These studies underscore the importance of static mechanical environments in controlling cellular processes.

In vivo, ECM mechanical properties are dynamic and predominantly governed by cellular remodeling, wherein cells degrade the matrix with MMPs or deposit and reorganize matrix proteins (7, 8). ECM degradability, defined as the rate at which cells enzymatically digest the matrix, significantly influences cell behavior. Studies suggest that degradation of ECM is a prerequisite for successful adhesion and force transmission from the cell to the ECM (9, 10). Moreover, ECM degradability affects stem cell differentiation, protein deposition, and the transition from single to collective modes of migration (11–13). In three-dimensional (3D) systems, degradability also pertains to the temporal evolution of space created by cellular MMPs, facilitating changes in cell shape, growth, and motility. Consequently, cells often exhibit enhanced spreading in degradable gels compared to nondegradable (ND) counterparts due to the physical confinement imposed by ND matrices (14).

Despite its evident role in 3D confinement, the impact of degradation rate on cell behavior in two-dimensional (2D) settings remains underexplored. Notably, Peng et al. (15) observed that human mesenchymal stem cells cultured on soft gels with higher degradability favored osteogenesis over adipogenesis, a process typically associated with stiff or stress-relaxing viscoelastic matrices (16). This observation suggests that cells may experience local temporal changes in tension on matrices with higher degradability, though this phenomenon has not been explicitly demonstrated. The prevailing paradigm posits that degradability and mechanical properties are uncoupled (17). Studies on elastic degradable gels have indicated that fibroblast-mediated remodeling alters the viscoelasticity of the environment (18, 19); however, disentangling the contributions of degradation versus matrix deposition remains challenging. It is unclear how cells perceive degradable environments post-MMP degradation and how this influences cellular tension.

Collagen, the most abundant protein in native ECM, has been extensively studied due to its critical role in development, regeneration, and various diseases (20). Unlike synthetic ECM systems, collagen comprises three polypeptide chains forming a triple helical structure (21), which assembles into fibrils and then bundles into fibers (22). MMPs specifically cleave collagen at sites located three-quarters of the length from the N-terminus (23), leading to unwinding of the hierarchical structure and further cleavage (24). Depending on the extent of degradation, this process can disrupt collagen crosslinks and induce microstructural alterations, such as changes in organization or reduced fibril thickness (25). However, the implications of these changes on fibrillar network mechanics are not well understood.

Crosslink disruption in both synthetic polymer gels and collagen fibril networks can increase polymer or fibril entanglements, potentially enhancing stress relaxation properties. We hypothesized that cells mechanically sense local ECM degradation through its effects on stress relaxation, a dynamic time-dependent mechanical property. To investigate this, we engineered collagen and synthetic hydrogels of varying degradability, subjected them to collagenase treatment, and assessed their postdegradation mechanics and cellular responses.

Our findings reveal that fibroblasts exhibit differential responses to collagenase-treated collagen matrices based on degradability. Rheology and colloidal probe atomic force microscopy (AFM) force relaxation measurements indicated that stress relaxation differences correlated with cellular responses. To isolate degradability as a tunable feature, we designed synthetic hydrogels incorporating MMP-degradable crosslinkers. By adjusting the ratio of degradable to ND crosslinks, we maintained consistent postdegradation stiffness across gels. AFM relaxation experiments confirmed alterations in stress relaxation following degradation in degradable gels, absent in ND counterparts. Fibroblasts cultured on these synthetic gels postcollagenase treatment displayed variations in spreading and focal adhesion formation, mirroring responses observed in collagen gels. Importantly, these responses were cell-type dependent. Extension of these studies to fibroblasts in 3D yielded comparable responses to the 2D conditions. Finally, we replicated our findings using a ND synthetic gel exhibiting faster relaxation characteristic of degraded matrices, further corroborating our results. Our study underscores the significance of degradability-mediated stress relaxation as a critical mechanical design parameter for future biomaterials.

## 1. Results

### 1.1. Collagen Degradability Tunes Cell Spreading in Response to Collagenase.

**Fig. 1.**
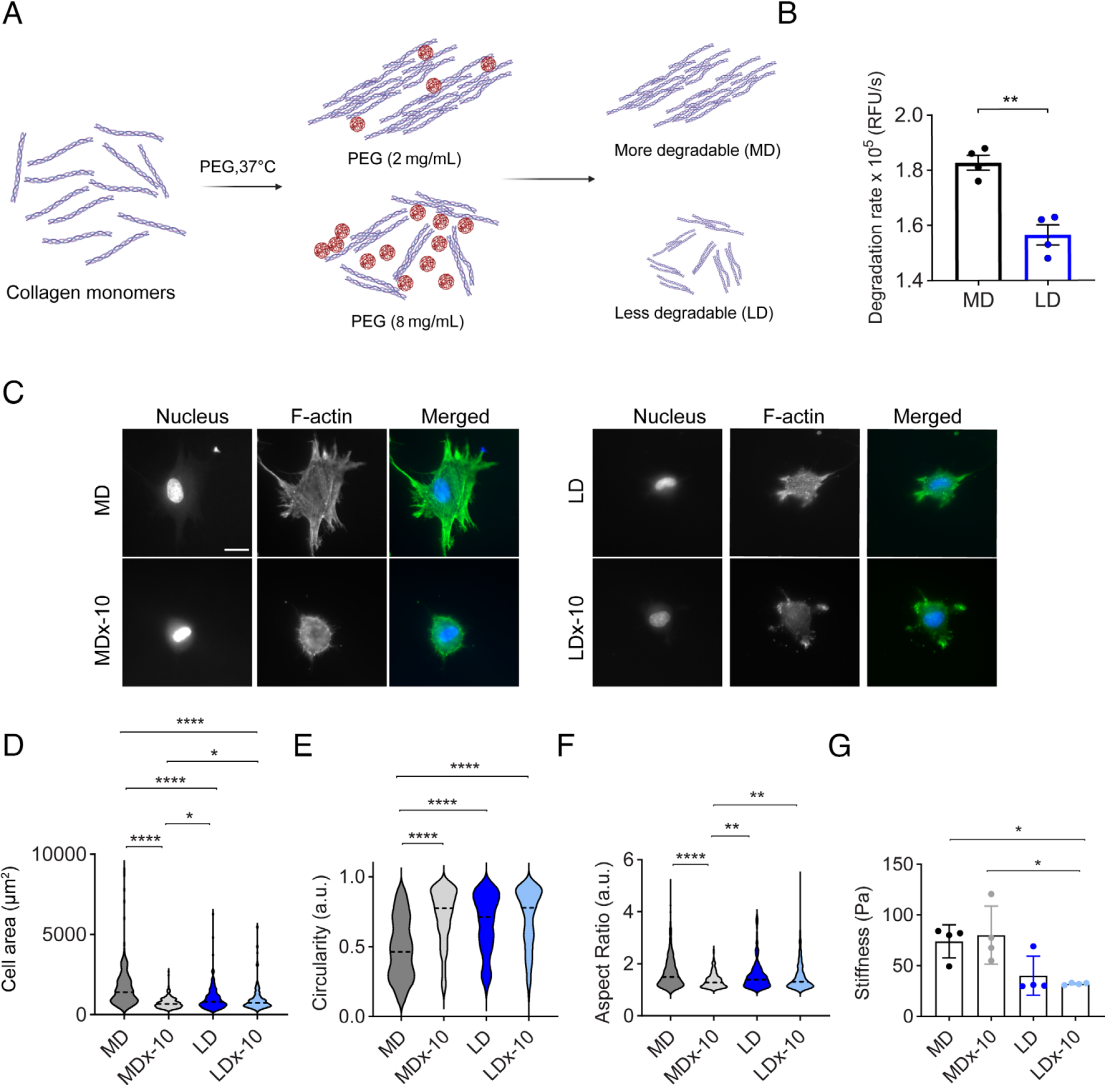
Influence of collagen degradability on cell spreading. MD - More degradable, MDx-10 - More degradable gels with 10 µg/mL collagenase treatment, LD - Less degradable, LDx-10 - Less degradable gels with 10 µg/mL collagenase treatment. (*A*) Schematic of process for producing collagen gels of varying degradability through macromolecular crowding with PEG (created with BioRender.com). (*B*) Degradation rate quantified by monitoring change in absorbance at 313 nm after adding collagenase, Student’s *t* test was performed between samples. (*C*) Representative fluorescence images of HFFs spreading on MD, MDx-10, LD, and LDx-10 gels. F-actin in green, nucleus in blue. (Scale bar represents 20 µm.) Quantification of HFF spreading: (*D*) area, (*E*) circularity, and (*F*) aspect ratio, n = 183 cells across N = 3 biological replicates. (*G*) Stiffness of gels measured using rheology across N = 4 independent gels. Statistical significance was determined using one-way ANOVA followed by the Tukey post hoc test, *P <0.05, **P < 0.01, and ****P < 0.0001.

To investigate how variations in matrix degradability influence cellular mechanosensing, we employed a macromolecular crowding technique to produce 2.5 mg/mL collagen gel substrates with distinct degradation profiles while maintaining consistent density (12) (Fig. 1*A*, *SI Appendix*, Fig. S1*A*). Specifically, we utilized 2 mg/mL and 8 mg/mL poly(ethylene) glycol (PEG) as a crowding agent with 2.5 mg/mL collagen to produce more degradable (MD) and less degradable (LD) gels, respectively. Postgelation, PEG was removed by washing (12). Degradation rates, assessed via absorbance at 313 nm following 10 µg/mL collagenase incubation, confirmed that MD gels degraded more rapidly than LD gels (Fig. 1*B*). These more degradable and less degradable collagen substrates serve as our initial model system. Treatment with 10 µg/mL collagenase, selected to mimic enzymatic degradation by cells, yielded the corresponding collagenase-treated gels denoted as MDx-10 and LDx-10.

Human foreskin fibroblasts (HFF), selected for their role as early responders in ECM remodeling (26–28), were cultured on each matrix condition to evaluate cellular responses to matrix degradation. After 4 h, intended to capture early cellular responses to degradation, we assessed cell spreading using metrics such as cell area, circularity, and aspect ratio. HFFs on MD gels exhibited an average spreading area of 1,741 µm^2^ with elongated morphologies, whereas those on LD gels displayed more rounded morphologies with a reduced average spread area of 1,007 µm^2^ (Fig. 1 *C*–*F*, *SI Appendix*, Fig. S1*B*). Collagenase treatment led to decreased spreading and increased roundness in cells on MDx-10 gels compared to MD gels (Fig. 1 *C*–*F*, *SI Appendix*, Fig. S1*B*). In contrast, cells on LDx-10 gels showed no significant differences in spreading, circularity, or aspect ratio compared to LD gels (Fig. 1 *C*–*F*, *SI Appendix*, Fig. S1*B*). These observations suggest that cells detect matrix changes mediated by degradation, potentially through alterations in mechanical properties. However, these changes only occur when the matrix is susceptible to degradation.

Rheological measurements indicated no significant stiffness differences between MD and MDx-10 gels, both averaging approximately 80 Pa (Fig. 1*G*). Similarly, LD gels before and after degradation exhibited comparable stiffness, averaging 30 Pa, which was lower than that of MD gels (Fig. 1*G*). AFM provided further characterization at the cellular scale (29, 30), revealing that MD gels were slightly stiffer than the LD gels, 62 ± 13 Pa versus 38 ± 18 Pa respectively (*SI Appendix*, Fig. S1*C*). Based on this, we hypothesized that MD gels may have thicker fibrils that increase fibril bending stiffness. Scanning electron microscopy (SEM) analysis showed that MD gels possessed thicker fibrils than LD gels, 0.13 ± 0.02 µm versus 0.097 ± 0.01 µm, respectively (*SI Appendix*, Fig. S1 *D* and *E*). Postdegradation, AFM measurements showed that MD gels demonstrated a reduction in stiffness from 62 ± 13 Pa to 30 ± 8.5 Pa, aligning with LD and LDx-10 gels, which remained unchanged (*SI Appendix*, Fig. S1*C*). SEM confirmed a decrease in fibril width in MDx-10 gels, 0.09 ± 0.008 µm, while LDx-10 gels showed no significant changes (*SI Appendix*, Fig. S1 *D* and *E*). In summary, bulk rheology suggested no stiffness differences between MD and MDx-10 gels, but local AFM measurements detected slight variations.

Given that stiffness alterations alone did not fully account for the observed cellular responses, we hypothesized that cells might be sensing differences in stress relaxation postdegradation. This led us to further investigate the role of stress relaxation as a potential mechanotransduction cue in degradable matrices.

### 1.2. Degradation Mediates Enhanced Stress Relaxation in Collagen Gels.

**Fig. 2.**
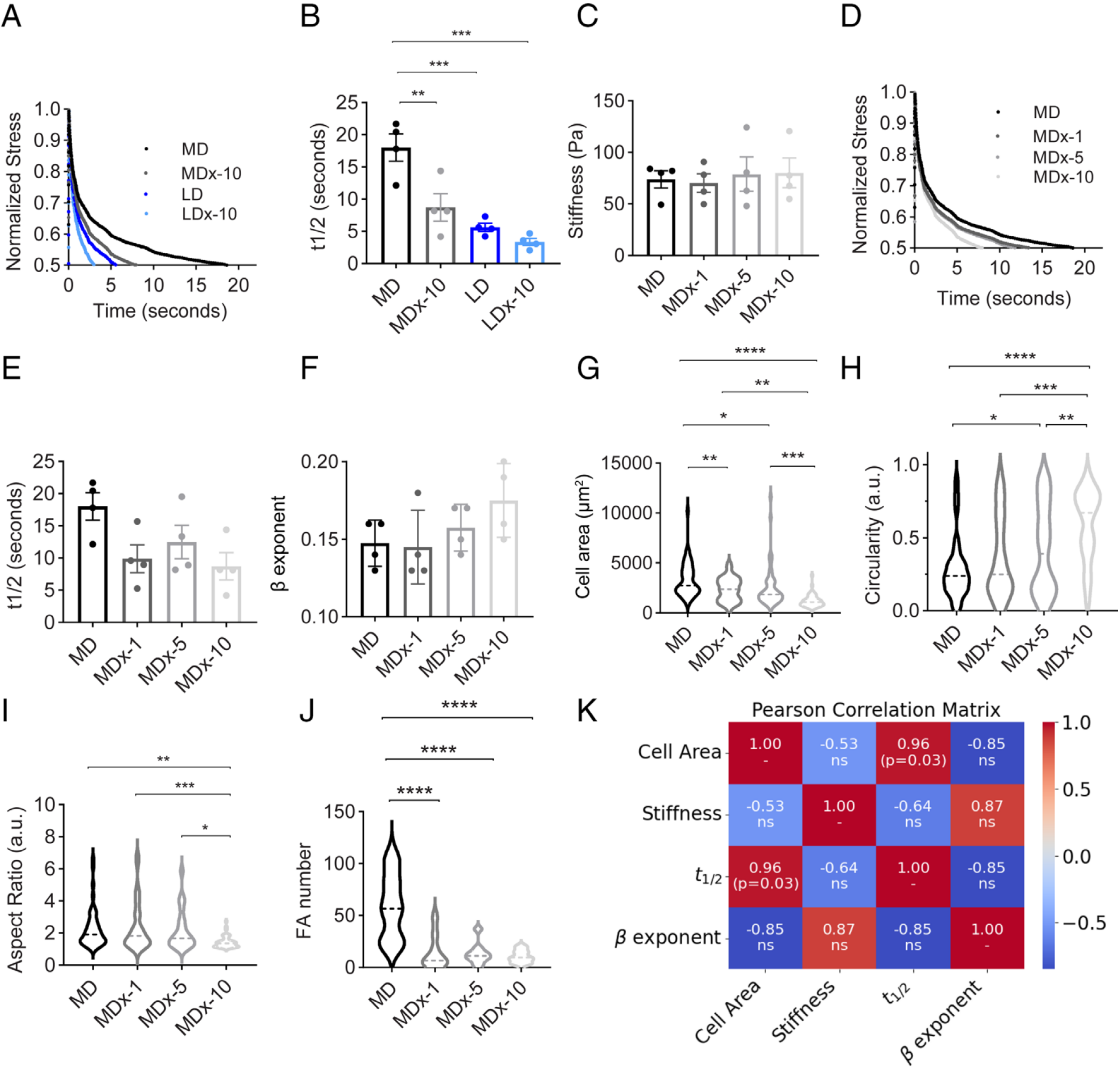
Relationship between degradability and stress relaxation of collagen. (*A*–*F*) Rheological measurements of collagen hydrogels. (*A*) Average stress relaxation curves of MD, MDx-10, LD, and LDx-10 gels. (*B*) Time taken for maximum stress to relax to one-half. (*C*) Stiffness of MD gels with different concentrations of collagenase treatment. MDx-1, 5, and 10 represent treatment with 1 μg/mL, 5 μg/mL, and 10 μg/mL, respectively. (*D*–*F*) Stress relaxation measurements on MD gels with different concentrations of collagenase treatment. (*G*–*I*) HFF spreading on collagen gels with different concentrations of collagenase treatment, n = 48 cells across N = 3 independent biological replicates. (*J*) Focal adhesion number per cell on MD, MDx-1, MDx-5, and MDx-10 gels with n = 16, 8, 9, and 16, respectively, across N = 3 independent biological replicates. (*K*) Pearson correlation matrix. Colors represent different correlation strengths, with blue and red representing strong negative and positive correlations respectively, N = 4 gels for the mechanical characterization, ns–no significance. Statistical significance was determined by one-way ANOVA followed by the Tukey post hoc test, *P <0.05, **P < 0.01, ***P < 0.001, and ****P < 0.0001.

To investigate how collagen gel degradability influences stress-relaxation, we conducted rheological measurements (*SI Appendix*, Fig. S2*A*). As shown in Fig. 2 *A* and *B*, MDx-10, LD, and LDx-10 gels exhibited enhanced relaxation compared to MD gels. The relaxation time to reach half the maximum stress (t_1/2_) was quantified, yielding 18.3 ± 3.7, 8.8 ± 4.5, 5.6 ± 1.3, and 3.4 ± 1.3 s for MD, MDx-10, LD, and LDx-10 gels, respectively. AFM-based stress relaxation measurements were also performed (*SI Appendix*, Fig. 2 *B*–*E*), but due to the rapid adhesion of the AFM tip to the collagen matrices in the DMEM environment, reliable measurements could not extend beyond 5 s. Details of AFM stress relaxation curve analyses are provided in *SI Appendix*, *Supplementary information* 2. Relaxation curves were fitted using the standard linear solid model (SLS) model and power-law rheology (PLR) models (*SI Appendix*, Figs. S3 and S4).

We next examined the concentration-dependent effects of collagenase treatment on MD gels by comparing intermediate treatment conditions (MDx-1 and MDx-5, corresponding to 1 μg/mL and 5 μg/mL collagenase) against MDx-10 (10 μg/mL collagenase) and untreated MD gels. Rheological measurements indicated no significant differences in stiffness among these conditions (Fig. 2*C*), but the average relaxation time t_1/2_ varied slightly (Fig. 2 *D*–*F*). HFFs cultured on these gels were analyzed for morphology, spreading, and focal adhesions. Compared to MDx-10 gels, cells on MDx-1 and MDx-5 gels showed increased cell spread area, reduced circularity, and increased aspect ratios, indicating concentration-dependent effects (Fig. 2 *G*–*I*). However, relative to MD gels, both MDx-1 and MDx-5 gels supported reduced spreading areas and focal adhesion numbers (Fig. 2 *G*–*J*, *SI Appendix*, Figs. S5 and S6). To identify the primary parameter affecting cell spreading, we constructed a Pearson correlation matrix comparing cell spread area, stiffness, t_1/2_, and beta exponent derived from the PLR model fits (Fig. 2*K*). Among these variables, t_1/2_ exhibited the strongest correlation with cell spreading area (r = 0.96, *P = 0.03).

To determine whether cell-endogenous proteolytic activity played a role in the cell spreading differences we observed at 4 h, we cultured HFFs on gels in the presence of a broad-spectrum protease inhibitor. Inhibition of endogenous MMPs did not alter the observed spreading differences, confirming that changes in stress relaxation were due to our exogenous collagenase treatment rather than further matrix degradation by cells (*SI Appendix*, Fig. S7).

Together, these findings demonstrate that collagen gel degradability translates into significant changes in stress relaxation after collagenase treatment. Moreover, t_1/2_ emerges as a strong predictor of cell spreading, underscoring the role of stress relaxation as a key mechanosensory cue in degradable matrices.

### 1.3. Design of Hydrogels with Varying Degradability Results in Tunable Stress Relaxation Post Collagenase Exposure.

**Fig. 3.**
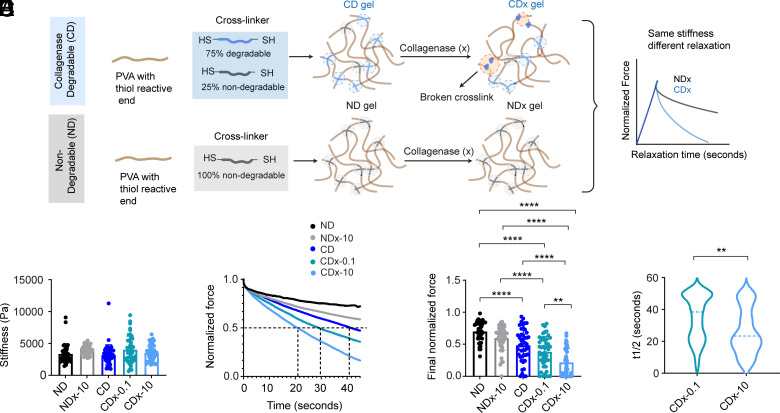
Rational design of synthetic PVA gels that stress relax as a function of degradability. (*A*) PVA gels of tunable degradability are generated by varying the ratio of ND to CD crosslinks (created with BioRender.com). (*B*) Stiffness of hydrogels measured using AFM, n = 45 across N = 3 independent samples, NDx-10 - ND treated with collagenase, CDx-0.1 - CD treated with 0.1 µg/mL of collagenase, and CDx-10 - CD treated with 10 µg/mL of collagenase. (*C*) Stress relaxation of hydrogels assessed using AFM. Dotted lines indicate the differences in relaxation time to reach half the max force for CD, CDx-0.1, and CDx-10. (*D*) Final normalized force at 45 s, n = 45 across N = 3 independent samples. (*E*) Time taken for the normalized force to relax to half of its maximum value. Statistical significance was determined using one-way ANOVA followed by the Tukey post hoc test, *P <0.05 and ****P < 0.0001, and Student’s *t* test **P < 0.01 respectively.

To emulate the effects we observed in our collagen model system, we aimed to engineer synthetic hydrogels such that degradability modulates stress relaxation. Poly(vinyl alcohol) (PVA) gels with tunable degradability were synthesized by varying the ratio of MMP-degradable to ND crosslinks. Two formulations were prepared: collagenase degradable (CD) and ND gels. The CD gels incorporated 75% CD crosslinks and 25% of ND crosslinks, while the ND gels comprised 100% ND crosslinks (Fig. 3*A*). AFM assessments confirmed that both CD and ND gels exhibited comparable stiffness, approximately 3.5 kPa (Fig. 3*B*).

Subsequent exposure of the gels to 10 µg/mL collagenase was conducted to evaluate degradation-dependent changes in mechanical properties. Control experiments with gels containing 100% MMP-degradable crosslinks demonstrated a significant reduction in stiffness postdegradation (*SI Appendix*, Fig. S8). However, the CD gels, with a combination of ND and degradable crosslinks, did not exhibit significant stiffness alterations following collagenase treatment (CDx-10), nor did the ND gels posttreatment (NDx-10) (Fig. 3*B*).

Next, we characterized the stress relaxation behavior of these hydrogels (Fig. 3*C*). For ND, NDx-10, and CD gels, over 70% of the relaxation data did not reach half of the maximum stress value within the experimental timeframe, indicating predominantly slow-relaxing behavior. In contrast, for CDx-10, percentages were closer to 10%, indicating fast-relaxing behavior (*SI Appendix*, Fig. S9), with CDx-10 gels achieving a t_1/2_ averaging around 25 s (Fig. 3*C*). CDx-10 gels also relaxed to a significantly lower normalized force at the experimental endpoint (Fig. 3*D*). Inspection of raw relaxation curves revealed that CDx-10 gels also exhibited relatively homogeneous behavior compared to NDx-10, CD, and ND gels (*SI Appendix*, Fig. S9), indicating more spatially uniform mechanical properties. PLR model fitting to the first 5 s of relaxation data indicated an increased power law exponent for CDx-10 gels, suggestive of increased viscous behavior (*SI Appendix*, Fig. S10). To achieve intermediate t_1/2_ values, CD gels were treated with varying collagenase concentrations. Treatment with 0.1 µg/mL of collagenase (CDx-0.1) resulted in an average t_1/2_ of approximately 35 s (Fig. 3*E*), demonstrating the tunability of relaxation properties through controlled enzymatic degradation.

In summary, our synthetic degradable hydrogels exhibit accelerated and enhanced stress relaxation following enzymatic degradation, while ND gels maintain slower relaxation profiles. In addition, the relaxation dynamics can be finely tuned by adjusting collagenase concentrations, providing a versatile platform for mimicking the mechanobiological properties of natural ECMs.

### 1.4. Fibroblasts Exhibit Less Focal Adhesions on Degradable Substrates After Collagenase Treatment.

Since HFFs responded to degradation-mediated stress relaxation of collagen gels, we hypothesized that they would exhibit a similar response on our synthetic gels. To facilitate cell attachment, RGD was conjugated to PVA prior to crosslinking. RGD-modified hydrogels showed no significant differences in stiffness or relaxation properties compared to their unmodified counterparts (*SI Appendix*, Fig. S11).

**Fig. 4.**
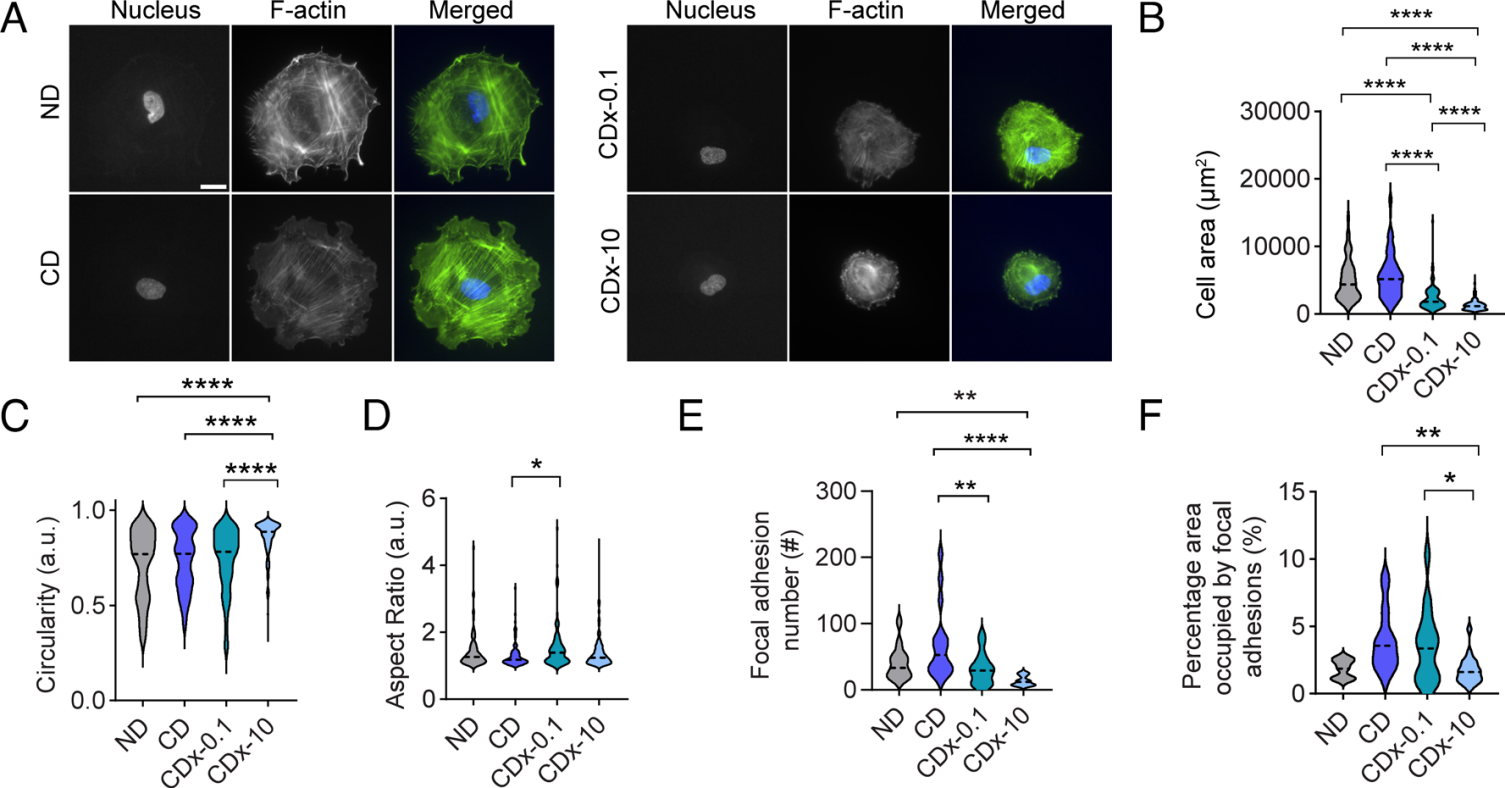
Fibroblast spreading on collagenase-treated degradable PVA gels. (*A*) Fluorescence micrographs of stained HFFs on gels. Actin (green) and nucleus (blue). (Scale bar, 20 µm.) (*B*) Quantification of HFF spreading area, (*C*) circularity, and (*D*) aspect ratio. N = 92 cells for ND and CD, N = 118 cells for CDx-0.1, and N = 247 cells for CDx-10 respectively across n = 3 biological replicates. (*E*) Number of focal adhesions per cell, based on vinculin staining, with area between 1 to 10 µm^2^. (*F*) Percentage of area occupied by focal adhesions per cell, based on vinculin staining. N = 13, 16, 15, and 21 cells analyzed for ND, CD, CDx-0.1, and CDx-10, respectively, across n = 3 biological replicates in panels *E* and *F*. For all graphs, statistical significance was determined using one-way ANOVA followed by the Tukey post hoc test, *P < 0.05, **P < 0.01, and ****P < 0.0001.

Initially, HFFs were cultured on CD and ND gels to evaluate their baseline behavior. As expected, no significant differences in the cell spreading area, circularity, or aspect ratio were observed between the two conditions (Fig. 4 *A* and *B*, *SI Appendix*, Fig. S12). HFFs spread extensively on both gel types, with mean areas of 5,004 µm^2^ and 5,676 µm^2^ on slow-relaxing ND and CD gels, respectively (Fig. 4 *C* and *D*). Focal adhesions in these conditions were generally punctate (Fig. 4 *E* and *F*, *SI Appendix*, Fig. S13), a hallmark of mature adhesion structures (31).

Next, we evaluated cell behavior on enzymatically treated gels. CDx-0.1 gels, with a t_1/2_ of 35 s, and CDx-10 gels, with a t_1/2_ of 23.5 s, were used to assess the impact of stress relaxation dynamics. On both degraded conditions, HFFs exhibited smaller spread areas and more circular morphologies compared to untreated CD gels (Fig. 4 *A*–*D*, *SI Appendix*, Figs. S12 and S14). Notably, cells on CDx-0.1 gels spread more extensively and were less circular compared to CDx-10 gels, consistent with the trend that faster relaxation limits cell spreading (Fig. 4 *A*–*C*).

Focal adhesion metrics further supported these findings. The number of focal adhesions per cell showed no significant differences between untreated CD and ND gels; however, both CDx-0.1 and CDx-10 gels exhibited reduced focal adhesion numbers compared to untreated CD gels (Fig. 4*E*, *SI Appendix*, Fig. S13). Similarly, the total area occupied by focal adhesions was diminished in CDx-0.1 and CDx-10 conditions relative to untreated CD gels (Fig. 4*F*).

Together, these results demonstrate that fibroblasts sense changes in stress relaxation induced by enzymatic degradation of synthetic hydrogels. Degraded synthetic gels exhibit faster relaxation dynamics and prompt reduced cell spreading and focal adhesion formation, mimicking the effects observed in degraded collagen gels.

### 1.5. Cell Response to Stress Relaxation Induced by Degradation is Cell Type Dependent.

To determine whether the response to degradation-enhanced stress relaxation is generalizable (32), we investigated two additional adherent cell types: C2C12 myoblasts and MCF10A mammary epithelial cells. Previous studies of the response of these cell types to substrate viscoelasticity have commonly used a 24 h culture period (33–36). Accordingly, we assessed cell spreading at 24 h on NDx-10 and CDx-10 gels.

#### 1.5.1. C2C12 myoblast response.

**Fig. 5.**
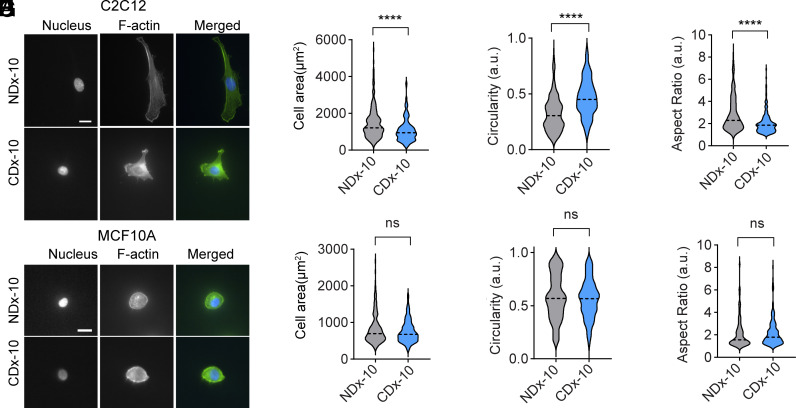
Spreading differences between cell types in response to collagenase treatment of degradable PVA gels. (*A*) C2C12 myoblasts spreading on NDx-10 and CDx-10 gels. Assessment of C2C12 spreading: (*B*) Area, (*C*) Circularity, and (*D*) Aspect ratio after 24 h of culture, n = 193 cells across N = 3 biological replicates. (*E*) MCF10A spreading on NDx-10 and CDx-10 gels. Quantification of MCF10A spreading: (*F*) Area, (*G*) Circularity, and (*H*) Aspect ratio after 24 h culture, n = 143 cells across N = 3 biological replicates. For all graphs, Student’s *t* test was performed, ****P < 0.0001.

We first examined C2C12 cells, which exhibited differential spreading behavior depending on substrate condition. Cells cultured on CDx-10 gels showed reduced cell spreading (mean area: 1,090 ± 650 µm^2^) compared to those on NDx-10 gels (mean area: 1,486 ± 877 µm^2^) (Fig. 5 *A* and *B*, *SI Appendix*, Fig. S15). Additionally, cells on CDx-10 gels were less elongated, with an average aspect ratio of 2.0 on CDx-10 gels compared to 2.9 on NDx-10 gels (Fig. 5 *C* and *D*). These results suggest that, similar to fibroblasts, myoblasts respond to degradation-enhanced changes in stress relaxation by modulating their morphology and spreading behavior.

#### 1.5.2. MCF10A mammary epithelial cell response.

Mammary epithelial cells are known to exhibit round morphologies on substrates with stiffness below 1 kPa and elongated morphologies on substrates with stiffness greater than 1 kPa (37). Consistent with these findings, MCF10A cells cultured on NDx-10 gels, which have a stiffness of approximately 3.5 kPa, displayed primarily well-spread and elongated morphologies (Fig. 5*E*, *SI Appendix*, Fig. S16). Interestingly, MCF10As on both CDx-10 and NDx-10 gels exhibited similar cell spread areas, circularity, and aspect ratios (Fig. 5 *F*–*H*) indicating that MCF10A did not respond to increased substrate viscoelasticity resulting from degradation.

These findings highlight cell type–specific responses to degradation-enhanced stress relaxation. While both fibroblasts and myoblasts exhibit reduced spreading and elongation on faster-relaxing CDx-10 gels, mammary epithelial cells appear insensitive to these changes in substrate viscoelasticity.

### 1.6. Fibroblasts in 3D Respond to Degradation–Induced Relaxation Similar to 2D.

**Fig. 6.**
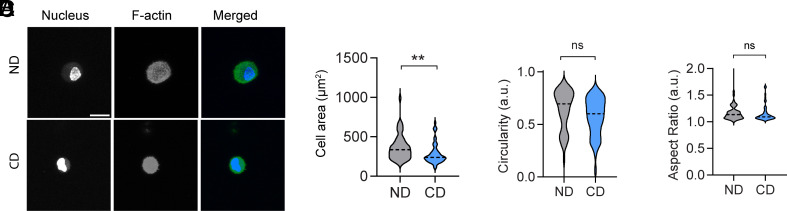
Fibroblast spreading in 3D degradable PVA gels. (*A*) 3D max intensity projections of HFF in ND and CD gels. (Scale bar represents 20 µm.) Assessment of HFF spreading: (*B*) Area, (*C*) Circularity, and (*D*) Aspect ratio after 4 h of culture, n = 50 cells across N = 3 biological replicates. For all graphs, Student’s *t* test was performed, **P < 0.01.

To advance the physiological relevance of our findings, we cultured HFFs in 3D, fully embedding them within CD and ND gels. We also omitted exogenous collagenases treatment to allow cell-mediated matrix remodeling via intrinsic MMPs. After a 4 h culture period, we quantified cell morphological parameters. HFFs encapsulated in ND gels exhibited a significantly larger mean spread area, 366 µm^2^, compared to those in CD gels, 284 µm^2^ (Fig. 6 *A* and *B*). However, no differences were observed in cell circularity or aspect ratio between the two conditions (Fig. 6 *C* and *D*). These results suggest that cell-mediated degradation in CD gels leads to stress relaxation effects analogous to those observed in our 2D studies, influencing HFF spreading behavior in a 3D context.

### 1.7. Fast Relaxing Poly(Acrylamide) Gels Promote Less Spreading Similar to Degradation–Induced Fast Relaxing Degradable Gels.

**Fig. 7.**
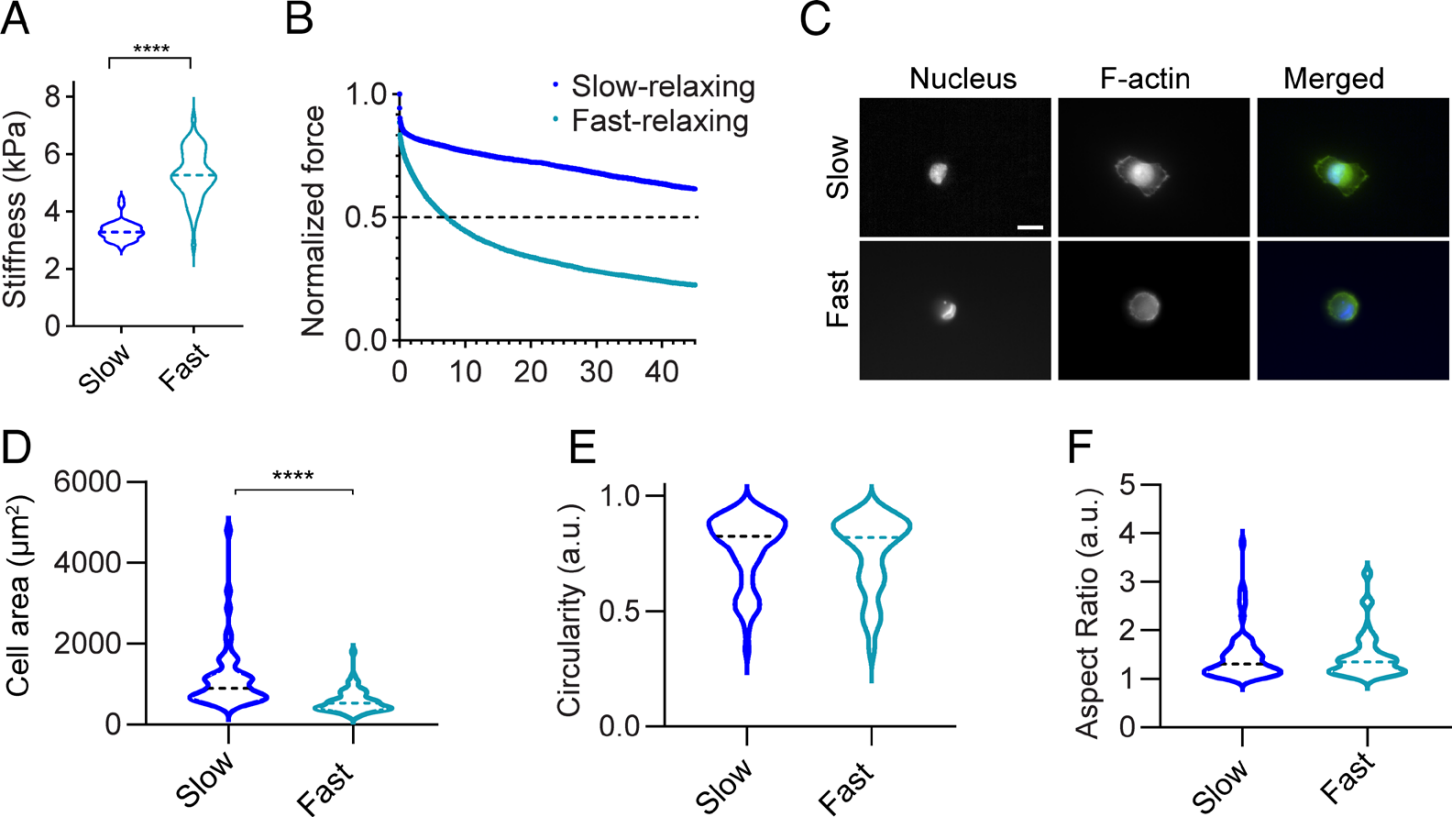
Fibroblast spreading in response to poly(acrylamide) gels of different stress-relaxing behavior. (*A*) Stiffness of elastic and viscoelastic formulation of gels measured using AFM. (*B*) Average stress relaxation curves of gels measured by applying 2nN force and monitoring relaxation for 50 s across, n = 45 force curves across N = 3 independent gels. (*C*) Cell spreading of gels on elastic and viscoelastic poly(acrylamide) gels. (*D*) Cell Area. (*E*) Circularity and (*F*) Aspect ratio, measured with n = 63 cells across, N = 3 biological replicates. For the statistical analysis, Student’s *t* test was performed, ****P < 0.0001.

To confirm that variations in cell spreading are attributable to changes in viscoelasticity postdegradation, we engineered poly(acrylamide) hydrogels with tunable relaxation times to emulate the mechanical behavior of degradable matrices. By adjusting the crosslinking density, we fabricated slow-relaxing and fast-relaxing gels of average stiffness 3.4 kPa and 5.3 kPa, respectively, as measured by AFM (Fig. 7*A*). Slow-relaxing gels did not relax to half of the maximum force, whereas fast-relaxing gels achieved a t_1/2_ of 14.8 ± 12 s (Fig. 7*B*). Similar results were obtained by rheology (*SI Appendix*, Fig. S17).

HFFs cultured on these gels showed marked differences in cell spread area. Cells on slow-relaxing gels exhibited significantly larger mean spread area, 1,117 µm^2^, compared to those on fast-relaxing gels, 555 µm^2^ (Fig. 7 *C* and *D*). However, no significant differences were observed in cell circularity or aspect ratio between the two conditions (Fig. 7 *E* and *F*). These findings show that cells respond similarly to changes in matrix viscoelastic properties, regardless of whether those properties are modulated through crosslinking density or degradation. They also highlight the crucial role that substrate stress relaxation plays in regulating cell behavior, independent of substrate stiffness.

## 2. Discussion and Conclusion

Both synthetic and native ECMs are often engineered to present precise elastic and viscoelastic mechanical cues to cells. Our study demonstrates that even slight degradation in such systems can alter these properties. Using AFM, we characterized cell scale-relevant changes in mechanical properties resulting from degradation and found that stress relaxation increased in both synthetic and collagen matrices. This change was readily sensed by cells: Fibroblasts and myoblasts responded to degradation-enhanced stress relaxation by spreading less and forming fewer focal adhesions on gels, whereas mammary epithelial cells did not exhibit such responses. As a control, fibroblasts cultured on a different synthetic ND hydrogel with varied relaxation times also spread less on fast-relaxing gels, despite the slightly higher stiffness of these gels compared to slow-relaxing ones. The consistency of fibroblast responses across collagen matrices and two synthetic hydrogel systems supports the conclusion that these cells sense degradation through its effect on matrix stress relaxation. Similar observations have been reported in previous studies. For instance, 3T3 fibroblasts cultured on poly(acrylamide) gels of varying viscous dissipation showed less spreading on substrates exhibiting enhanced relaxation (38). Conversely, fibroblasts have been shown to spread more on stress-relaxing alginate substrates compared to their elastic counterparts, depending on ligand concentration (27). Additionally, alginate matrices with enhanced stress relaxation promoted C2C12 myoblast cell spreading and elongated morphologies (34), which contrasts with our observations. However, in the prior study, C2C12 cells were cultured on elastic alginate gels with little relaxation and compared to slow stress-relaxing gels with t_1/2_ > 120 s, whereas our NDx-10 gels and CDx-10 gels exhibited average t_1/2_ of 43 and 23.5 s, respectively. Thus, the slow-relaxing matrices from Bauer et al. (34) are similar to our NDx-10 gels, which did promote extensive cell spreading and high aspect ratios.

To investigate the generalizability of our results to more physiological conditions, we also performed 3D culture of fibroblasts in ND and CD gels, where degradation was dependent on endogenous cellular MMPs. Cell spreading was diminished in 3D CD gels, similar to collagenase-degraded CD gels in 2D. Our findings contradict previous research showing that both degradability and viscoelasticity increase cell spreading in 3D alginate gels (27, 39). One possible explanation is that ionic crosslinking between alginate chains results in ligand clustering, locally increasing adhesion ligand density and promoting more spreading (27). Another potential reason is that the nanoporous architecture of our hydrogels may limit cell spreading and mechanosensation (40). Previous studies evaluated cell spreading after several days of culture, while we evaluated after 4 h of culture. Further investigation into time-dependent changes in hydrogel architecture is warranted. Indeed, recent work showed that soft ND matrices promoted enhanced spreading of mouse embryonic fibroblasts on day 1 but less spreading on day 14 when compared to stiff degradable matrices (41). In future studies, it will also be important to evaluate how primary cells respond to degradation-enhanced stress relaxation, as our study only evaluated immortalized cells.

Cell response to substrate viscoelasticity is known to depend on cell type and the dynamics of relaxation (42). For instance, Mandal et al. (32), utilized stress-relaxing poly(acrylamide) gels to assess the spreading behavior of normal hepatocytes and hepatocellular carcinoma cells. While normal hepatocytes showed less spreading on stress-relaxing substrates, carcinoma cells exhibited enhanced spreading compared to elastic substrates of the same stiffness. In our study, mammary epithelial cells did not respond differently to degradation-enhanced fast relaxing CDx-10 substrates, t_1/2_ of 23.5 s, in 2D at 24 h. A recent study that cultured mammary epithelial cells on poly(acrylamide) gels of 2 kPa stiffness with varying viscous dissipation showed cell spreading was inhibited on highly dissipating matrices, similar to our findings with fibroblasts (36). However, other research demonstrated that mammary epithelial cell spheroids exhibited enhanced spreading in 3D fast-relaxing substrates, t_1/2_ of 30 s, compared to slow-relaxing substrates, t_1/2_ of 350 s (43). The inconsistency of these results may be attributed to differences in ligand density, which has been suggested to modulate the viscoelastic contribution to spreading (27, 34). Another important factor to consider is the characterization technique, i.e. bulk (compression or rheology) vs. local AFM, and the method of force application (shear vs. compression), which complicates direct comparisons of relaxation timescales. We argue that cell-scale-relevant characterizations are needed to gain a deeper understanding of physiologically relevant timescales. In summary, cellular responses to stress relaxation appear to vary, even among cells of the same type, depending on relaxation dynamics, assessment methods, and factors like ligand density.

Enzymatic degradation of ECM is known to reduce stiffness (44); however, we found that it can also increase stress relaxation independent of stiffness reduction. The extent and nature of mechanical changes is likely to depend on the concentration and duration of enzyme exposure, enzyme activity levels, and the specific degradation mechanism. Consequently, the matrix mechanics experienced by cells in native and synthetic degradable ECMs depend on the matrix’s susceptibility to cell-mediated degradation and the cell’s capacity to produce degrading enzymes. This also means that degradability can be engineered to present cell- and MMP-specific stress relaxation responses over time. Tuning the architecture of degradable crosslinkers could offer additional control over enzyme binding affinity, thereby regulating degradation-enhanced stress relaxation times. Moreover, the type of adhesion ligand can modulate cellular responses to viscoelasticity (38). Future studies should investigate how specific adhesion ligands, such as GFOGER, RGD, or IKAV, influence cellular responses to degradation-enhanced stress relaxation.

To modulate collagen gel degradability in our study, we employed a previously developed approach utilizing macromolecular crowding. Notably, degradability correlated with macromolecular crowding-induced changes in collagen architecture: More degradable gels exhibited thicker fibers that became thinner after degradation compared to less degradable gels. While elucidating the mechanisms underlying this difference in degradability and fibril network mechanics is beyond the scope of this work, we speculate that collagen fibril thickness regulates both degradability and the subsequent viscoelasticity of collagen matrices. Indeed, our recently published multiscale model predicted that matrix microarchitecture and fibril thickness influence the degradability of collagen matrices (45).

In vivo, ECM degradation plays pivotal roles in maintaining tissue homeostasis, and its misregulation is involved in diseases like fibrosis, arthritis, and cancer (46). This duality is often understood as a balance between MMP activity and matrix deposition. However, our study highlights additional critical factors: the susceptibility of the ECM to degradation and its effects on stress relaxation and cell adhesion. Our findings also support the emerging concept of a relationship between fibrillar matrix architecture and degradability (12, 45), which could provide insight into why certain matrix architectures correlate with distinct cellular behaviors (12, 47–49). For example, fibril architecture-mediated differences in degradability may underlie the increased stress relaxation observed in advanced glycan end products-crosslinked collagenous tissues (50). Similarly, comparisons of stress relaxation behavior and ECM architecture between wild-type mice and those with MMP-resistant collagen-I could yield valuable insights, as mutant mice exhibit markedly different outcomes in tumor studies (51, 52). Investigating whether these relationships extend to other fibrous ECM, such as fibrin, will also be critical.

In conclusion, our findings demonstrate that even minimal exposure of hydrogels to degrading enzymes, without significant alteration in stiffness, can profoundly impact stress relaxation behavior. This work leverages degradability as a design variable to engineer stress relaxation and cellular adhesion, with far-reaching implications for the development of future biomaterials. For instance, hydrogel degradability could be tailored to specific cell types or MMP activity to spatially and temporally modulate stress relaxation properties. This work also underscores the dynamic nature of cellular interactions with degradable materials. Local degradation by cells can result in materials that are biophysically distinct from their initial characterizations. Since biomaterial properties are typically assessed postmanufacture without accounting for cellular modification, adjusting for degradation effects is crucial for engineering biomaterials that sustain desired environmental conditions and regulate cell behavior effectively. This consideration is particularly important in regenerative medicine, where degradable scaffolds are used to promote cell infiltration and tissue repair.

## 3. Experimental Section

### 3.1. Chemicals.

3-D Life PVA-PEG kit hydrogel kit SG (Cat. No.:G82-1), 3-D Life PVA-CD kit hydrogel kit SG (Cat. No.:G83-1) and 3-D Life RGD peptide (Cat.No.: 09-P-001) were purchased from Cellendes GmbH.

High concentration, rat tail acid extracted type I collagen was procured from Corning (Corning, NY). PEG (8000 Da) was ordered in powder form from Sigma-Aldrich (St Louis, MO) and reconstituted in PBS (Life Technologies, Carlsbad, CA) immediately before usage with a final concentration of 100 mg/mL.1 × reconstitution buffer composed of sodium bicarbonate, HEPES-free acid, and nanopure water.

### 3.2. Preparation of Collagen Hydrogels with Different Architectures.

First, PEG of required amounts to make a 2 or 8 mg/mL final concentration (denoted as P2 or P8) was added to DMEM. This is followed by addition of the reconstitution buffer and mixing well. Thereafter, collagen stock was added to the mixture to produce a final concentration of 2.5 mg/mL. Finally, pH of the final mixture was adjusted using 1 N NaOH, followed by incubation at 37 °C (∼45 min). Following polymerization, PEG was washed out of the gels by rinsing with DMEM 3 × for 5 min each. The gels were then degraded for 40 min using 10 μg/mL of clostridium histolyticum collagenase from Sigma Aldrich (St Louis, MO) followed by rinsing with DMEM for 3× times.

### 3.3. Preparation of Synthetic Hydrogels with Tunable Degradability.

Gel consisting of 5 mM of thiol-reactive PVA (molecular weight in range 40 to 300 kDa as per manufacturer) was formed using 3-D Life hydrogels according to the recipe shown in Table 1. For the gel preparation, the components listed in Table 1 are added in sequence. For cell culture, 0.5 mM of RGD is incubated with PVA for 20 min at room temperature followed by the addition of the remaining components in Table 1 followed by mixing. After adding the crosslinker, the mixed solution was sandwiched between hydrophobic coverslip (DCDMS coated) and glutaraldehyde-treated coverslip and allowed to react at 37 °C for 45 min. The ND crosslinker consists of PEG while the CD crosslinker consists of PEG attached to a degradable peptide sequence (Pro-Leu-Gly-Leu-Trp-Ala).

**Table 1.** Preparation of hydrogels with tunable degradability

Component	ND gel	Degradable gel (CD)
Water	15 μL	15 μL
10 x CB (7.2)	3.75 μL	3.75 μL
PVA	7.5 μL	7.5 μL
DMEM	7.5 μL	7.5 μL
CD-Link	-	8.43 μL
PEG-Link	11.25 μL	2.82 μL

The ND and CD gels were then treated with 10 μg/mL of Type I *Clostridium* histolyticum bacterial collagenase for 5 min and then washed with DMEM for 3× times followed by characterization.

### 3.4. AFM on Gels.

AFM was used to characterize the local change in the mechanical properties of the hydrogels. AFM experiments were performed using a MFP-3D Bio atomic force microscope (Oxford Instruments) mounted on a Ti-U fluorescent inverted microscope (Nikon Instruments) using the force contact mode. Gold-coated silicon nitride probes with 12 μm diameter borosilicate glass particle attached (Product: PT.GS, Novascan, USA), of nominal spring constant 0.03 N/m, were used for the measurements. The probes were calibrated using the thermal noise method in DMEM followed by indenting on a glass surface in DMEM for calibrating the deflection sensitivity using the built in Asylum Research Software (Igor Pro, Wavemetrics).

The gels were fabricated on the glutaraldehyde-coated coverslips using the procedure outlined in a previous work (53) to ensure covalent attachment of gels during AFM indentations performed in liquid (DMEM). The thickness of the hydrogels produced was measured to be greater than 100 μm. For the stiffness measurements, the approach curve was fitted with a Hertz model for a spherical indenter geometry using the Asylum Research software which is described as follows (54):

$$F=\frac{4}{3}\frac{E}{\left(1-v^{2}\right)}R^{1/2}\delta^{3/2},$$

where F is the indentation force, R corresponds to the radius of the indenter, E is the Young’s modulus, and v is the Poisson’s ratio which is assumed to be 0.5 for elastic materials and δ is the indentation depth. The fits were performed on the first 35% of the force curve to quantify the Young’s modulus of the gels.

We sandwiched the pregel solution between the glutaraldehyde-coated coverslip and a dichlorodimethylsilane (DCDMS) coated coverslip for the synthetic gels. For the collagen gels, the gels were casted onto glutaraldehyde-coated coverslips without a hydrophobic coverslip cover. The thickness of the synthetic gels was around 150 µm for the synthetic gels and the collagen gels were around 400 µm. The diffusion coefficient of collagenase was estimated at approximately 7.4 × 10^−7^ cm^2^/s (55). The time to diffuse through 400 µm collagen gels was calculated to be around 36 min. Hence, we treated the collagen gels for 40 min. On the other hand, the synthetic gel’s thickness was calculated to be around 130 to 150 µm. We calculated the time to diffuse through was around 5 min. Hence, we used 5 min for the synthetic PVA gels. The AFM characterization was performed immediately after degradation and washing for both the collagen gels and the synthetic PVA gels. For the poly(acrylamide) gels, the AFM characterization was performed after allowing the gels to swell overnight to replicate the overnight incubation with adhesion ligands that precedes cell culture.

For the force-relaxation experiments on synthetic gels, an indentation speed of 2 μm/s was used to achieve a relative trigger of 2 nN followed by holding the z-position of the probe for 50 s in which the force changes were captured before the probe was retracted. At least 15 viscoelastic measurements per sample were performed at random locations in the sample spaced at least 100 μm apart between locations. The viscoelastic measurements in which the approach curves were noisy were excluded from the analysis. For each condition, at least three individual gels were characterized. In the degradable gels, some of the relaxation curves completely relaxed within 50 s of experimental acquisition time, and reached small negative values as the forces have completely relaxed. In such cases, the final normalized force value was taken as zero.

### 3.5. Preparation of Poly(Acrylamide) Gels with Tunable Relaxation.

Poly(acrylamide) gels were prepared by mixing acrylamide (40% w/v stock solution) monomers with (2% w/v stock solution) bis-acrylamide crosslinkers. Ammonium persulfate (APS, 10% w/v stock) and tetramethylethylenediamine (TEMED) were used as initiator and catalyst. For the elastic slow-relaxing gels, acrylamide and bis-acrylamide with final concentrations 5% (w/v) and 0.15% (w/v) were mixed and degassed for 15 min. For the fast-relaxing gels, final concentrations of 35% (w/v) of acrylamide and 0.01% (w/v) of bis-acrylamide were used. To the degassed mix, 10 μL of APS and 1 μL of TEMED per mL was added to initiate polymerization. 25 μL of this gel mixture was then added to a hydrophobic coverslip made by adding a few drops of dichlorodimethylsilane to a glass coverslip. The gel mixture was then covered using a glutaraldehyde-coated coverslip. After 1 h of polymerization, the glutaraldehyde coverslip was carefully removed and incubated with (4-(2-hydroxyethyl)-1-piperazineethanesulfonic acid) (HEPES) buffer for 30 min.

### 3.6. Functionalization of Poly(Acrylamide) Gels.

After incubation in HEPES buffer, 1 mg/mL of sulfosuccinimidyl 6-(4′-azido-2′-nitrophenylamino)hexanoate (sulfo-SANPAH) and kept under 365 nm UV light for 10 min. The gels were then washed with HEPES buffer and replenished with sulfo-SANPAH again followed by exposure to 365 nm UV light again. Cyclo(-RGDfK) (Aladdin Scientific, USA) of 0.5 mM was then added on top of the gels and left at 4 °C overnight. The gels were then incubated in 70% of ethanol for 30 min. Finally, the gels were washed thoroughly with DMEM three times and used for cell culture.

### 3.7. Rheology.

Rheology was performed using an ARES-G2 rheometer equipped with a 20 mm parallel plate geometry. To prevent slipping of hydrogels, 25 mm glass coverslips functionalized with glutaraldehyde were attached to the top geometry and the bottom plate. For the stress relaxation experiments, a 5% strain was applied followed by monitoring the relaxation for 50 s. Stiffness of the gels was measured at t = 0 in stress relaxation experiments. In addition, frequency sweep experiments were also performed to determine storage and loss modulus between 0.05 to 10 Hz of frequency.

### 3.8. Relaxation Curve Analysis.

The relaxation curves were analyzed by fitting either the SLS model or the PLR model using nonlinear least squares fit. For the SLS model, the normalized force-relaxation curves were fitted using the following equation (56):

$$F\left(t\right)=F_{\infty }+\left(F_{0}-F_{\infty }\right)e^{-t/\tau },$$

where F_∞_ refers to the final long-term equilibrium force, F_0_ refers to the instantaneous force and τ represents the relaxation time.

For the PLR model, the relaxation curves were fitted using the following equation (56):

$$F\left(t\right)=F_{0}{\left(1+t/t^{\prime }\right)}^{-\beta },$$

where F_0_ refers to the instantaneous force, t′ represents small time offset which does not affect relaxation behavior, β represents the power law exponent.

For the PVA gels, PLR model was applied for the first 5 s of relaxation data (*SI Appendix*, Fig. S9).

For some of the CDx-0.1 measurements, the relaxation did not cross half of maximum force within the timespan of experiment and for these measurements the t_1/2_ was taken as 50 s (total timespan of experiment).

The final relaxation amplitude or the final normalized force is determined by averaging the last 5 s of the relaxation data.

### 3.9. SEM.

SEM was performed on the hydrogels using FEI SEM Apreo equipped with an ETD detector. Both the collagenase-treated gels and the nontreated gels were first fixed with 4% paraformaldehyde for 1 h. This is followed by 3× rinsing in PBS for 10 min of each step of shaking. The gels were then rinsed twice with Milli-Q water for 15 min each. The fixed gels were then subjected to a series of dehydration steps in ethanol and hexamethyldisilazane (HMDS) using the protocol outlined in ref. 57. Briefly, the gels were dehydrated first in ethanol dilution series: 30, 50, 70, 90, and 100% for 15 min of each step. The hydrogels were then incubated in ethanol/HMDS dilution series: 33, 50, 66, and 100% for 15 min each. After the final incubation, the gels were allowed to dry in an aluminum foil for at least 1 d in the fume hood. The dried gel samples were then sputter coated with a Pelco SC-7 sputter coater with gold as the target. The gels were then imaged at 5 kV and 0.6 nA with 15,000x magnification. At least 12 images were acquired per sample, across three replicates for the quantification of mean width. For the width analysis, we used SIMpoly software (57, 58).

### 3.10. Cell Culture.

HFF (passages 11–24), C2C12 (passages 3–10), and MCF10A (passages 7–14) cell lines were purchased from ATCC. HFF and C2C12 were cultured in high glucose Dulbecco’s modified Eagle’s medium (DMEM) supplemented with 10% (v/v) fetal bovine serum (FBS, Corning, Corning, NY) and 0.1% (v/v) gentamicin (Gibco Thermo Fisher, Waltham, MA). For the MCF10A cells, growth media consisted of DMEM/F12 (Gibco, Waltham, MA) supplemented with 5% horse serum, 20 ng mL-1 epidermal growth factor (EGF), 0.5 mg mL-1 hydrocortisone, 100 ng mL-1 cholera toxin, 10 µg mL-1 insulin and 0.1% (v/v) gentamicin. The cells were maintained at 37 °C and 5% CO_2_ in a humidified environment during culture and imaging. The cells were passaged every 2 to 3 d as required. Cells were trypsinized and spun down and cultured on top of both synthetic and native collagen gels after washing 3× DMEM.

For the MMP inhibitor studies, HFFs were pretreated with 10 µM of GM6001, a broad spectrum protease inhibitor, for 30 min before culturing them on the hydrogels in the presence of GM6001.

### 3.11. Focal Adhesion Staining and Quantification.

The focal adhesions were quantified using vinculin staining. For vinculin staining, fixed samples were blocked using 2% BSA for 2 h at room temperature and then incubated primary vinculin monoclonal antibody (1:250, V9131, Sigma) for 2 h. Following incubation, samples were rinsed 3× times in PBS to remove unbound primary antibodies. Then, samples were incubated with a secondary antibody conjugated with alexa-546 for 1 h at room temperature followed by imaging after 3× times rinsing in PBS.

For the quantification of focal adhesions, we adopted a modified procedure from Tolbert et al. and Horzum et al. (59, 60) Briefly, the images were background subtracted using rolling ball radius to 2 pixels in Fiji (61). The local contrast of the image is enhanced by applying the CLAHE (Contrast limited adaptive histogram equalization) plugin with set parameters as follows: blocksize = 19, histogram bins = 256, maximum slope = 3. We then applied Gaussian blur with a sigma radius of 2 followed by a Mexican hat filter with sigma radius = 2. The filtered images were binarized using the Otsu thresholding method. Finally, using the Analyze particles option we selected circularity from 0.00 to 0.99 and size above 1 µm^2^. The focal adhesion number reports size ranges 1 to 10 µm^2^ and the area per focal adhesion was calculated by dividing the total area of focal adhesions divided by the cell area obtained from the phalloidin staining.

### 3.12. Cell Area Quantification.

For the HFFs in both 2D and 3D conditions, after 4 h of culture, cells were fixed in 4% paraformaldehyde and then stained with DAPI and phalloidin for quantifying the cell area. For C2C12 and MCF10A cell lines, fixing and staining were performed after 24 h.

The cell area, circularity, and aspect ratio were obtained using the images stained with phalloidin and DAPI. Trackmate-Cellpose/Fiji plugin (62) was used for the analysis. The DAPI channel was used to confirm individual cells. The phalloidin green channel was used for the actual analysis. Quantification was performed by running cyto2 (cytoplasm 2.0) pretrained model on the images of phalloidin-stained cells, with an approximate cell diameter value provided as the input parameter.

For the analysis in Fig. 1, cells that were in contact with each other were also included. For HFF culture with different concentrations of collagenases in Fig. 2, only cells interacting with the matrix were included, while cells in contact with other cells were excluded.

For the 3D cell spreading analysis, z-stacks were obtained using a 40x (NA: 1.1) Leica TCS SP8 inverted confocal microscope with 1 µm step size. The maximum intensity projection of the z-stacks was used for calculating the cell area, circularity, and aspect ratio.

## Supplementary Material

Appendix 01 (PDF)

## Data Availability

All study data are included in the article and/or *SI Appendix*.
