# Supplementary material for: Matrix degradation enhances stress relaxation, regulating cell adhesion and spreading
Source: Proc Natl Acad Sci U S A. 2025 Mar 25;122(13):e2416771122. doi: 10.1073/pnas.2416771122 (PMC12002262; doi:10.1073/pnas.2416771122)
Supplement: Supplementary file 1 — Appendix 01 (PDF) [file pnas.2416771122.sapp.pdf]

Supporting information

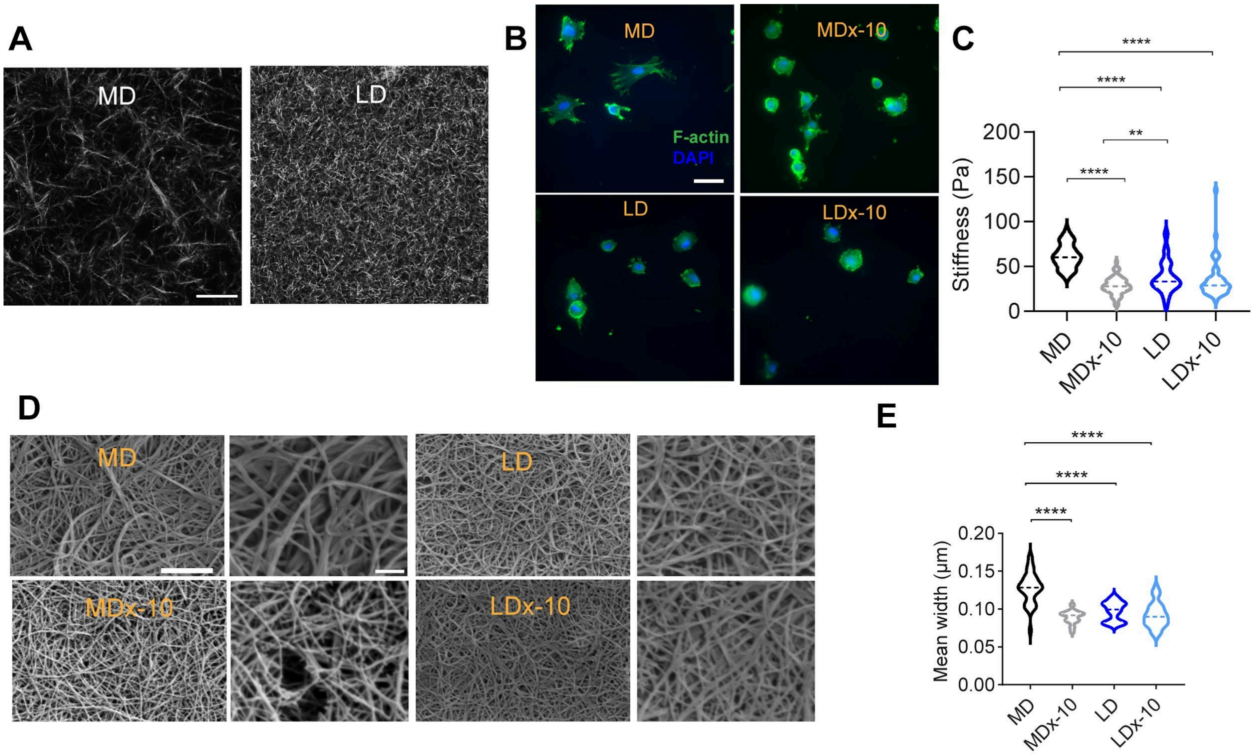

Figure S1. (A) Confocal images of MD and LD gels. Scale bar represents 20 µm. (B) Representative images of HFF on MD, MDx-10, LD and LDx-10 gels in one field of view. (C) Stiffness of gels measured using AFM. Statistical significance was determined using ANOVA, $^{**}p < 0.01$, and $^{****}p < 0.0001$. (D) Scanning electron micrograph images of MD, MDx, LD and LDx gels, imaged at 15,000x and 30,000x magnification. Scale bar represents 2 µm for the left panel and 500 nm for the corresponding right panel. (E) Mean widths obtained from SEM images. The data show values obtained from at least 12 images obtained from three independent samples.

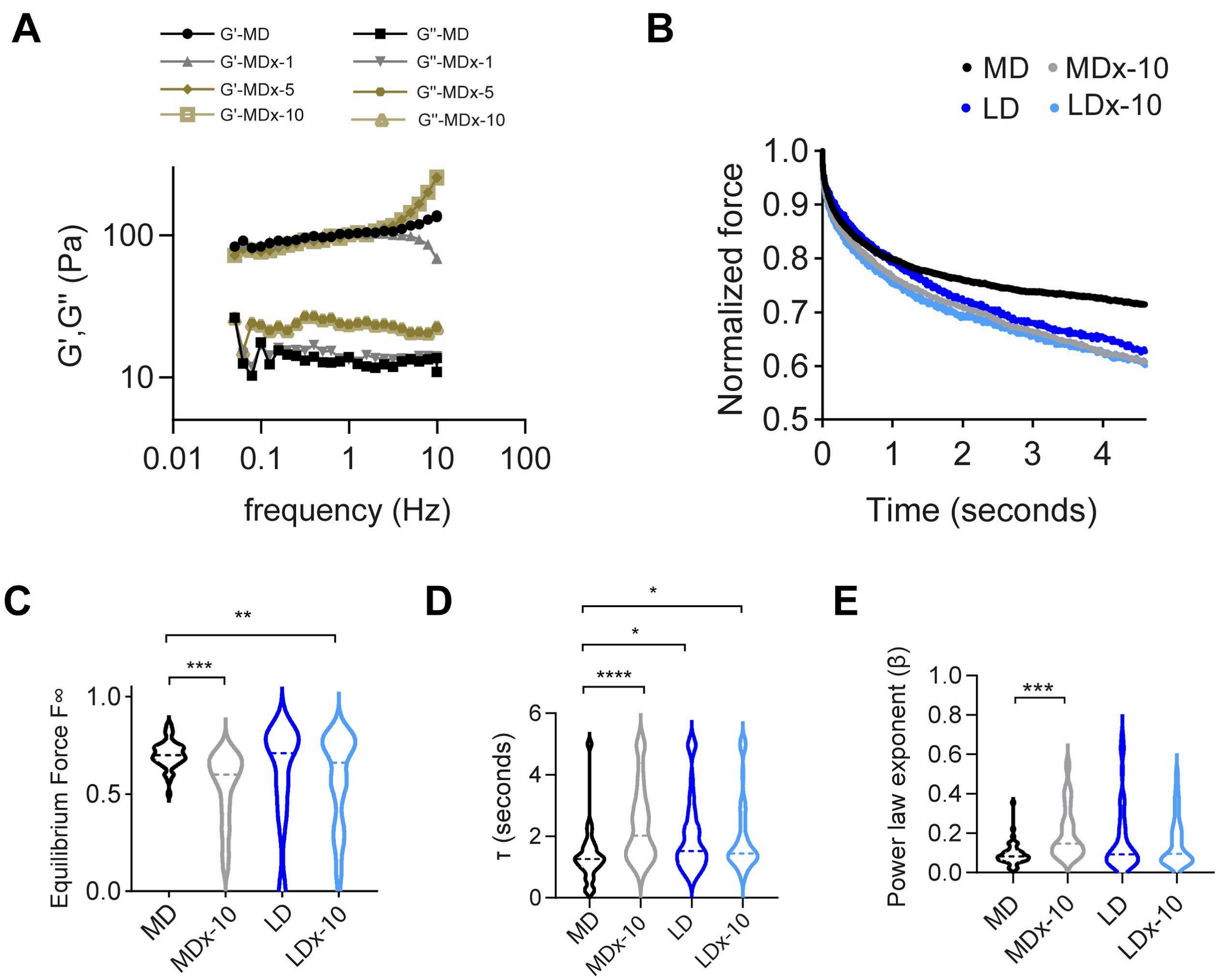

Figure S2. Differences in degradability of collagen gels correspond to differences in stress relaxation. MD - More degradable, MDx-1, MDx-5 and MDx-10 - More degradable gels with collagenase treatment of 1, 5 and 10 µg/mL respectively, LD - Less degradable, LDx-10 - Less degradable with collagenase treatment. (A) Frequency sweep of collagen hydrogels with different concentrations of collagenases. (B) Average of relaxation curves. (C) Standard linear solid parameter F_∞. (D) Parameter T relaxation time. (E) Power law rheology fit parameter β. The data are obtained from n=3 independent samples, N = 15 measurements per sample. For all graphs, statistical significance was determined by ANOVA, $^*p < 0.05$, $^{**}p < 0.01$, $^{***}p < 0.001$, and $^{****}p < 0.0001$.

Supplementary information 2:

AFM measurements showed that MDx-10 gels exhibited greater stress relaxation compared to MD gels (Figure S2B). On the other hand, LD gel relaxation behavior was similar to LDx-10 gels and MDx gels (Figure S2B). These data show that changes in stress relaxation correlate with matrix degradation. Moreover, enhanced stress relaxation (LD, LDx-10, MDx-10) correlates with less cell spreading (Figure 1D-F). To further quantify the stress relaxing behavior of the gels, we

fitted the standard linear solid (SLS) model to the relaxation curves (Figure S2C,D). The SLS model characterizes relaxation using equilibrium force (long-term) and relaxation time. Between the MD and LD gels, we observed no difference in the equilibrium force (F_∞) (Figure S2C). However, the relaxation time τ showed an increase for LD indicating that these gels are slightly more viscous in nature (Figure S2D). For the MDx-10 gels compared to their non-degraded counterpart (MD), an obvious increase in τ and a decrease in F_∞ was seen. Specifically, the average F_∞ and τ for MDx gels were 0.54 and 2 seconds, respectively, compared to 0.7 and 1.4 seconds for the MD gels (Figure S2C, S2D). LDx-10 gels compared to LD gels did not show any difference in either of the SLS parameters. Overall, the SLS model reveals an increase in both F_∞ and τ for the MD gels after degradation, which are similar to the F_∞ and τ values of LD and LDx-10 gels, and may explain the observation of less cell spreading MDx-10, LD, and LDx-10 gels.

The SLS model failed to capture the initial time points ($t < 0.1$ s, Figure S3), which is consistent with previous experiments on cells(56). Therefore, we also performed a power law rheology (PLR) model fit (Figure S2E) to the relaxation curves. This yielded better fits than SLS (Figure S4). Higher values of the beta exponent in PLR corresponds to enhanced relaxation and more fluid-like behavior. The beta exponent showed an increase from an average of 0.09 to 0.2 for MDx-10 gels compared to MD gels, suggesting a more fluid-like behavior post-degradation. Similar to the SLS fits, PLR fits showed that LD and LDx-10 gels did not exhibit differences in their beta exponents.

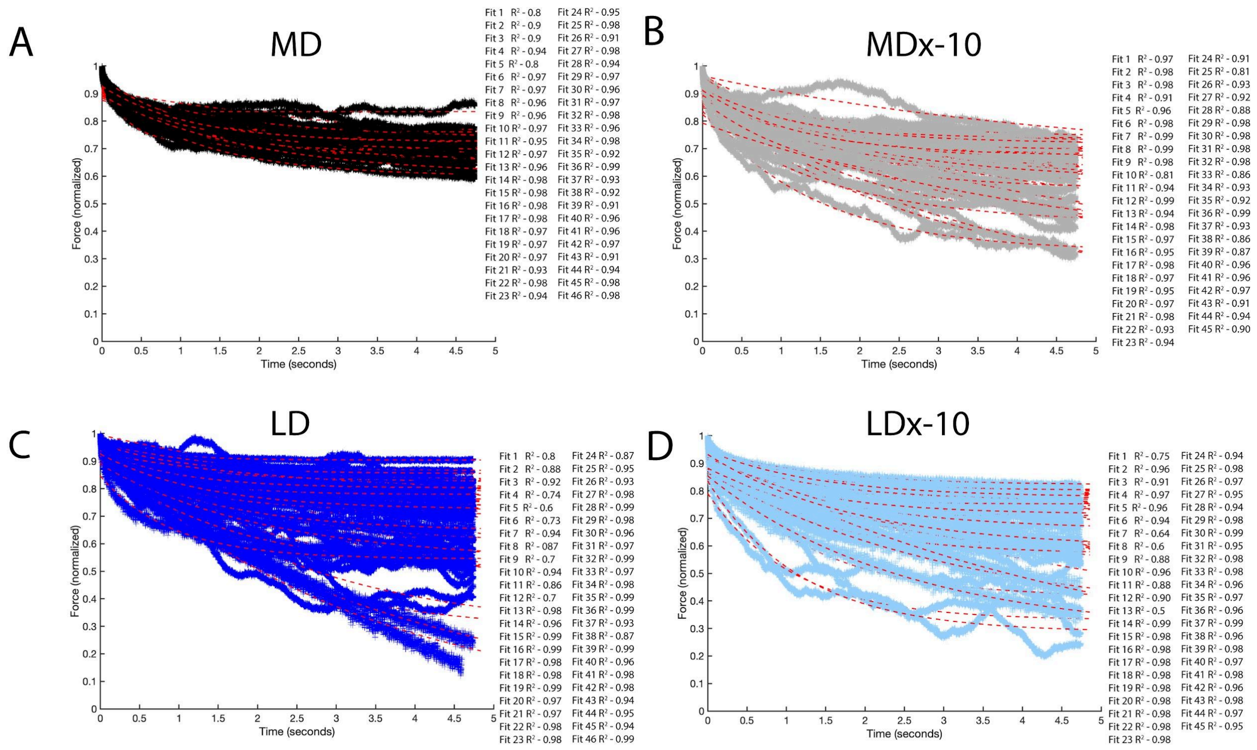

Figure S3. Standard linear solid fits for collagen gels.

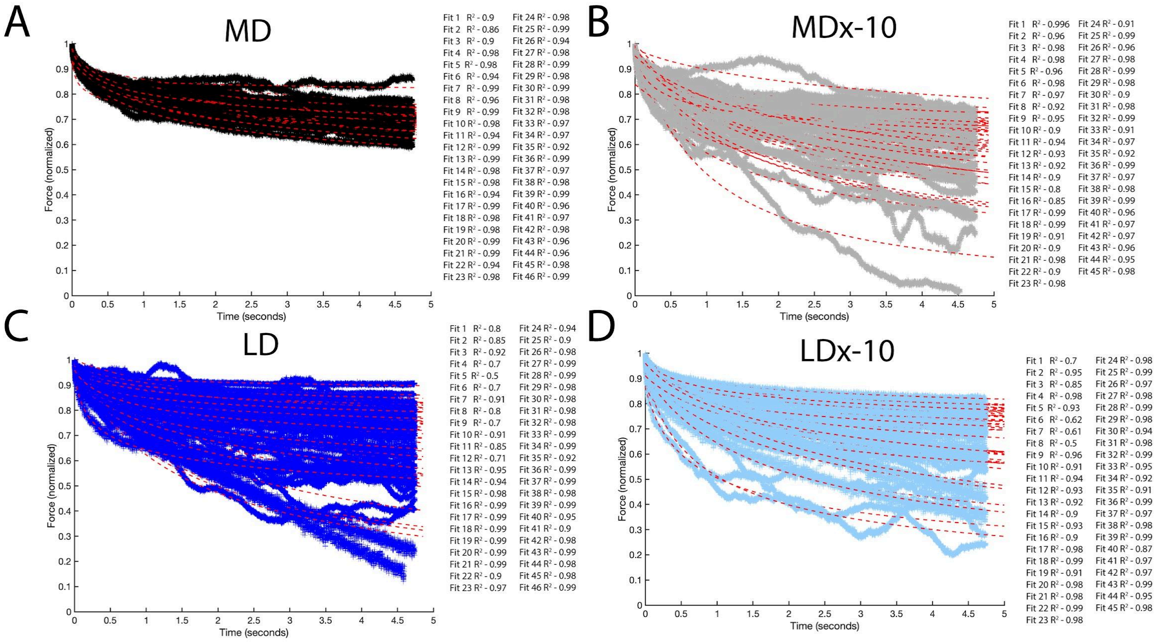

Figure S4. Power law fits for collagen gels.

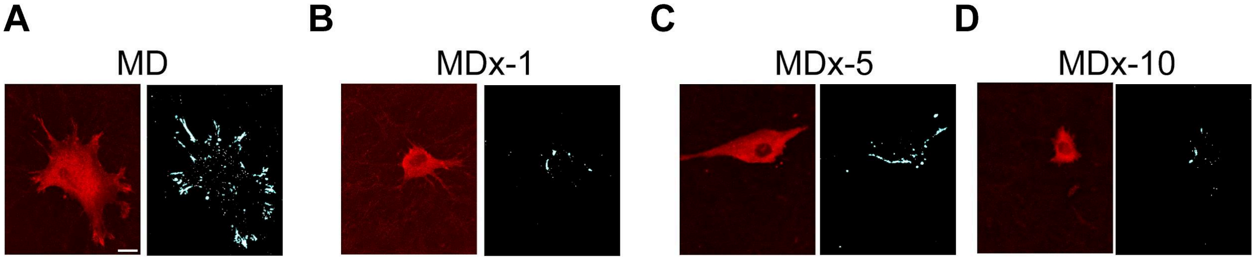

Figure S5. Representative images of vinculin stained cells and the corresponding processed images for focal adhesion quantification on collagen gels. (A) MD (B) MDx-1 (C) MDx-5 and (D) MDx-10. Scale bar represents 20 μm.

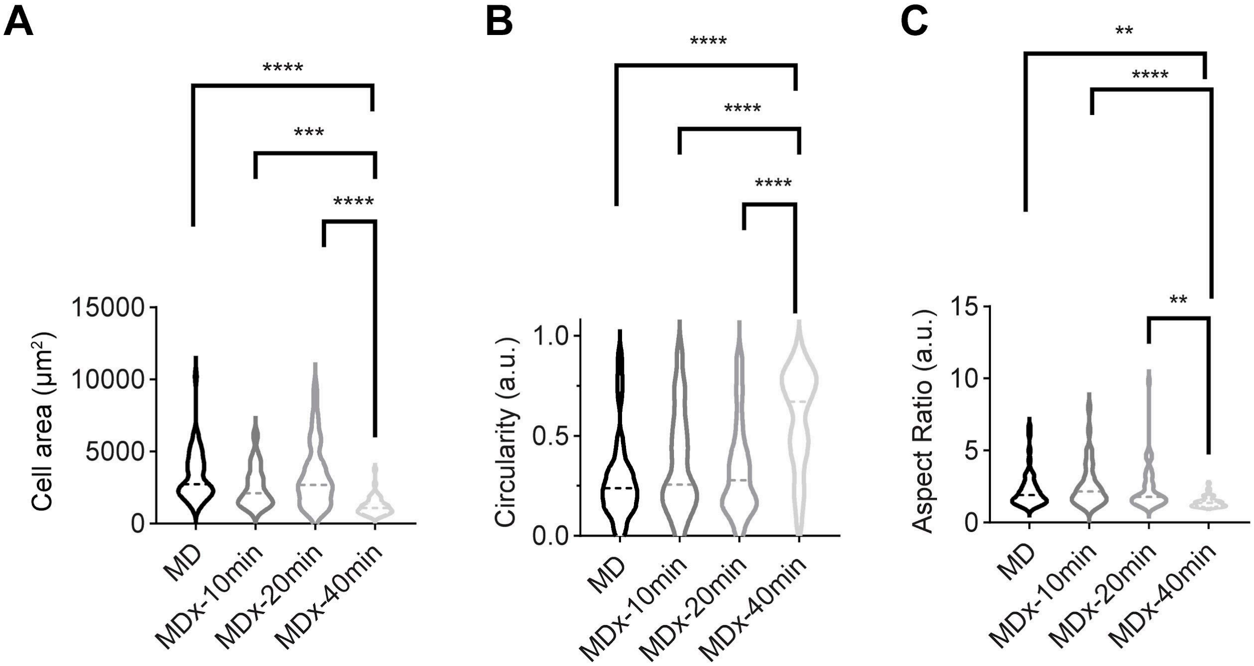

Figure S6. HFF spreading with different time treatments of 10 μg/mL of collagenase (B) Cell area (C) Circularity and (D) Aspect ratio, n=48 cells analyzed across three biological replicates. Statistical analysis were performed using ANOVA, **$p<0.01$, ***$p<0.001$, and ****$p<0.0001$

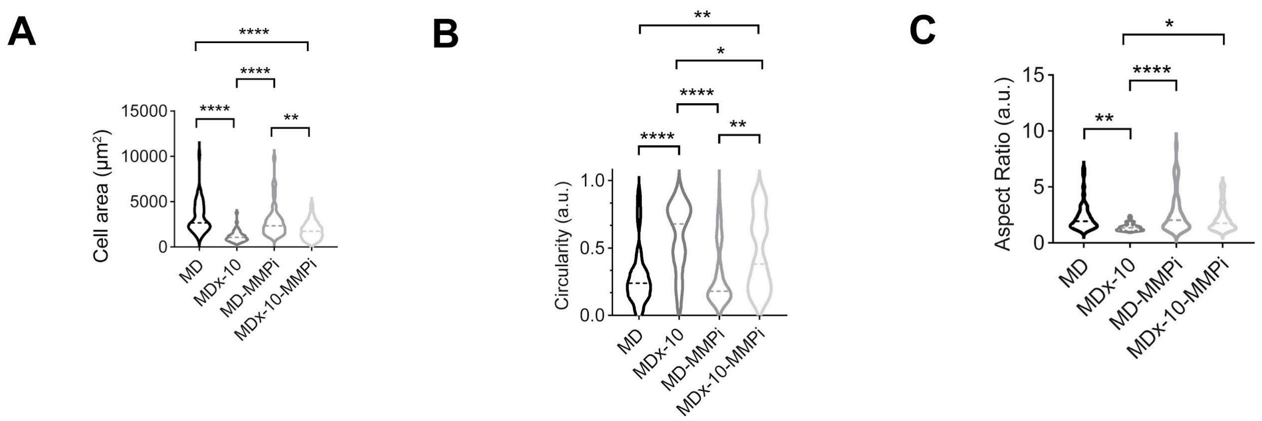

Figure S7. Assessment of HFF spreading in the presence of 10 μM concentration of GM6001. HFF spreading on collagen gels. (A) Cell area (B) Circularity and (C) Aspect ratio. Statistical analysis were performed using one-way ANOVA across n=48 cells from N=3 biological replicates, *p<0.05, **p < 0.01, and ****p<0.0001.

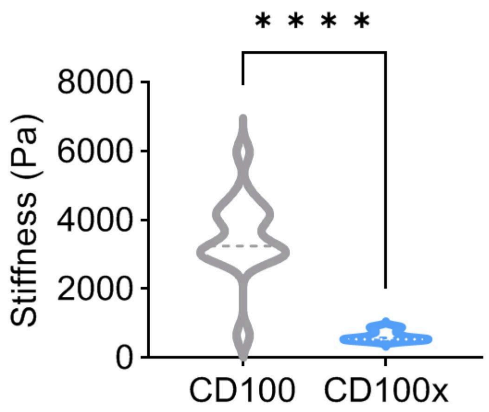

Figure S8. Stiffness of PVA gels made with 100% degradable crosslinks. The gels exhibited significant reduction in stiffness after treatment with collagenase.

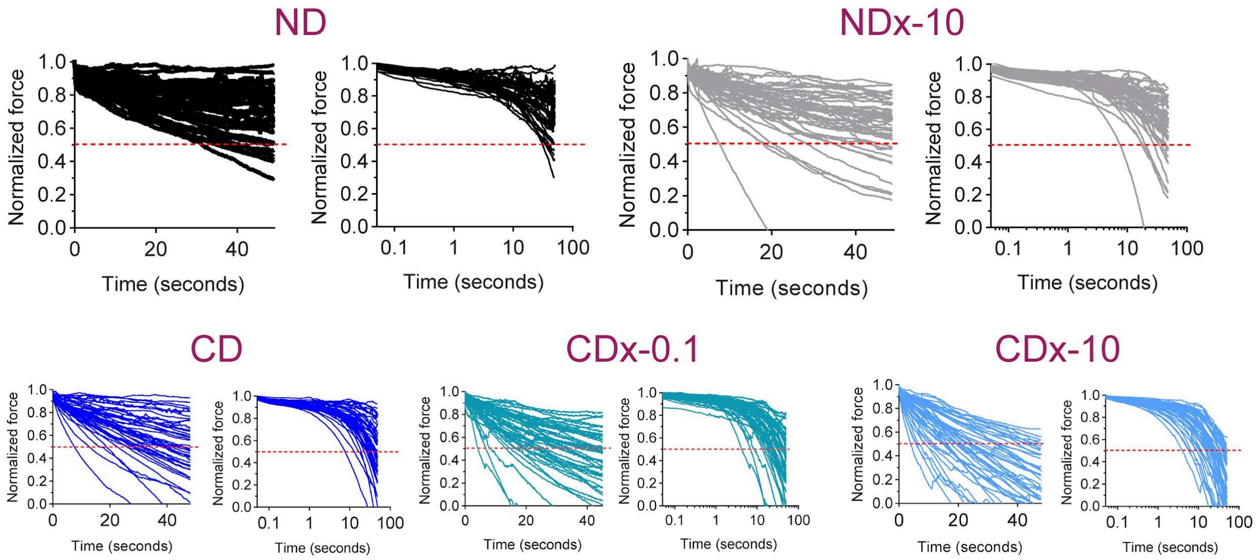

Figure S9. Raw relaxation curves of ND, NDx-10, CD, CDx-0.1 and CDx-10 with normalized force with time axis in linear and log-scale.

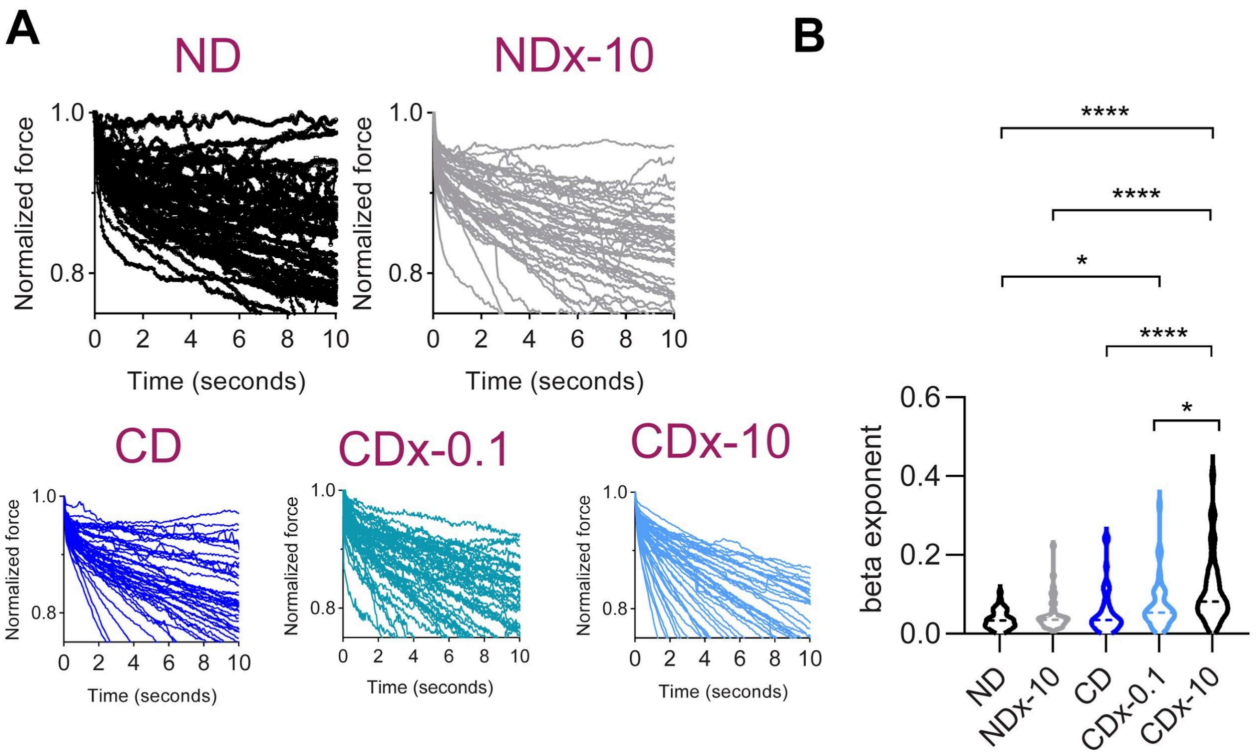

Figure S10. (A) First 10 seconds of raw relaxation curves . (B) Power law rheology fits to the first 5 seconds of PVA gel data. One-way ANOVA analysis, ****P<0.0001.

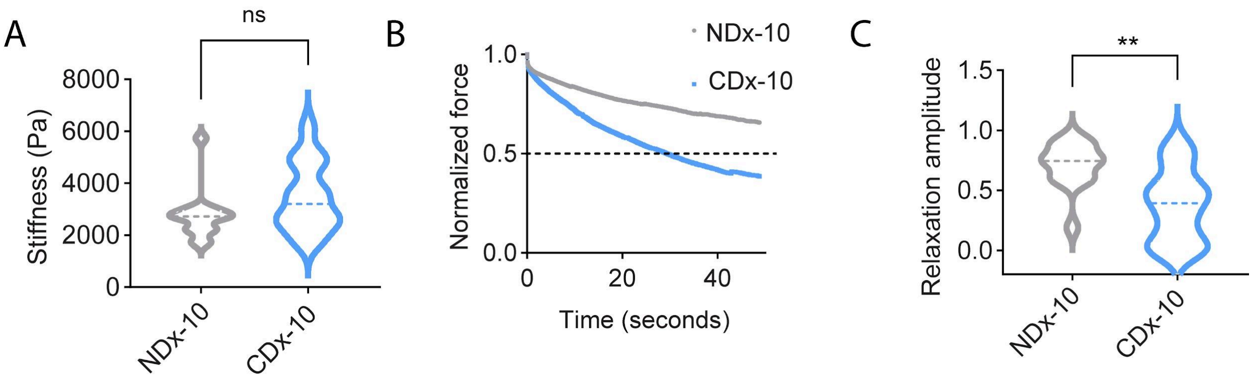

Figure S11. RGD modified hydrogels. (A) stiffness. (B) Average relaxation curve. The $t_{1/2}$ was calculated to be around 25 ± 14 seconds. Three of the curves in CDx gel did not relax to more than half of the maximum value. None of the NDx-10 gels reached 0.5 in the experiments. (C) Relaxation amplitude across data, **P < 0.01. N=15 measurements.

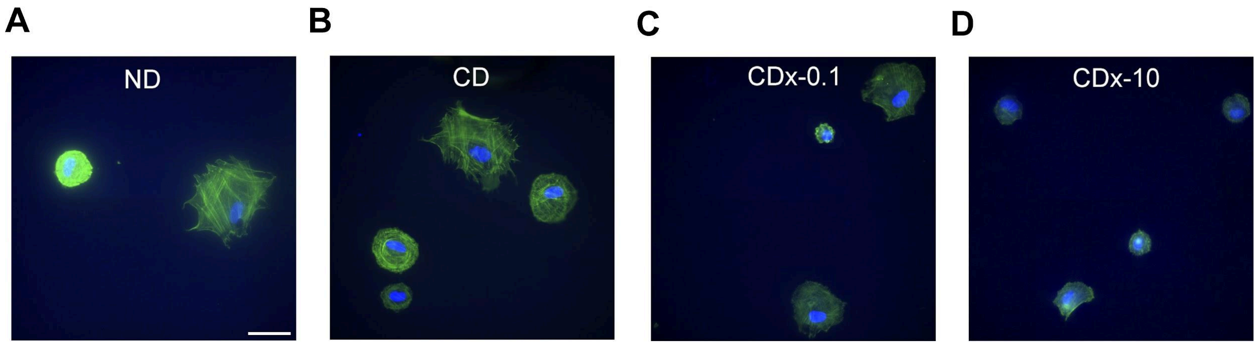

Figure S12. Images of cells in one field of view for human foreskin fibroblasts. (A) ND (B) CD (C) CDx- 0.1 and (D) CDx-10. Scale bar represents 50 μm.

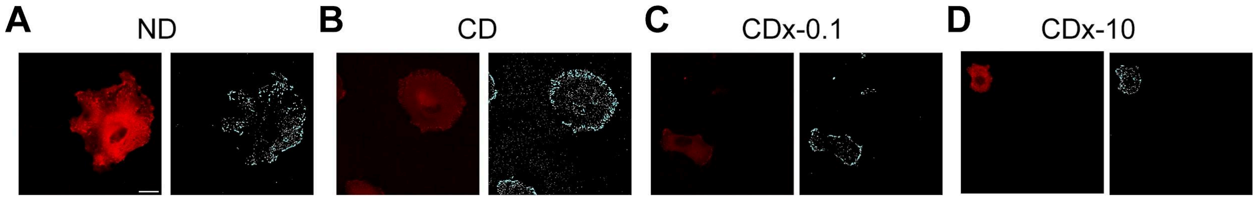

Figure S13. Representative images of vinculin stained cells and the corresponding processed images for focal adhesion quantification on PVA gels. (A) ND (B) CD (C) CDx-0.1 and (D) CDx-10. Scale bar represents 20 μm.

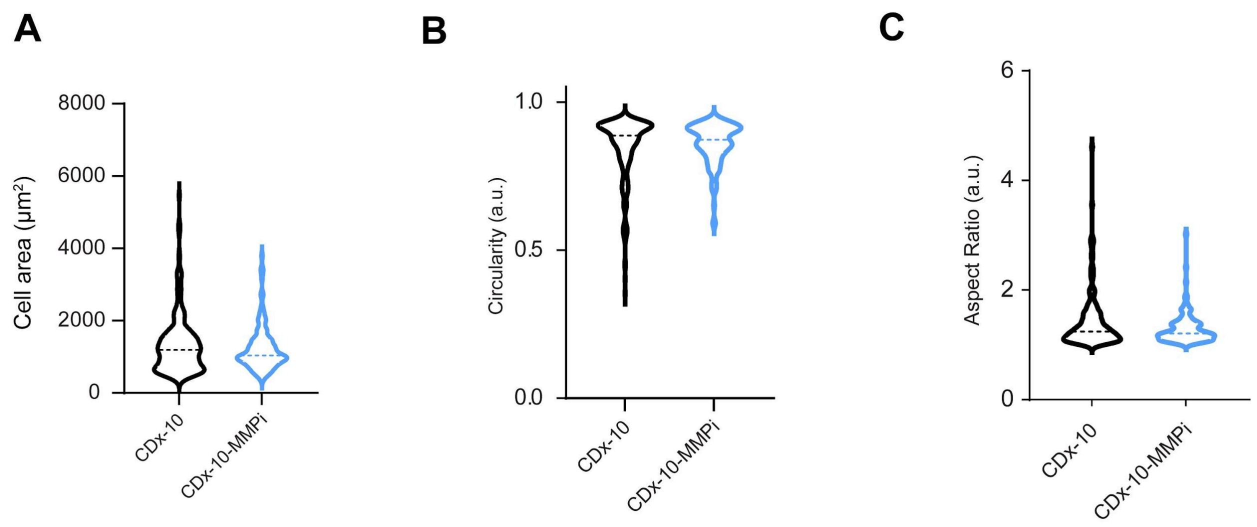

Figure S14. Assessment of HFF spreading in the presence of 10 μM concentration of GM6001 on PVA CDx-10 gels. HFF spreading on MMP inhibited degradable CDx-10 gels. n=247 cells for CDx-10 gels and n=131 cells for CDx-10-MMPi across N=3 biological replicates. (D) Cell area. (E) Circularity and (F) Aspect ratio. Statistical analysis was carried out using the student's t-test.

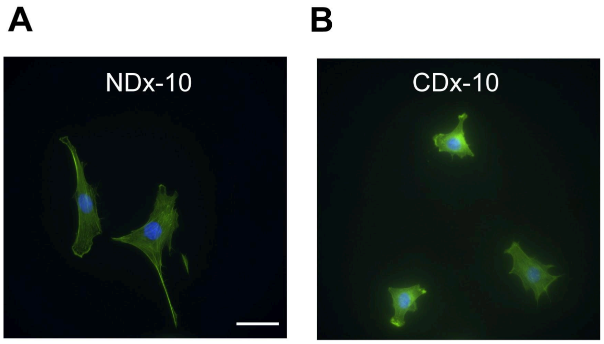

Figure S15. Images of cells in one field of view for C2C12 myoblasts. (A) NDx-10 (B) CDx-10. Scale bar represents 50 μm.

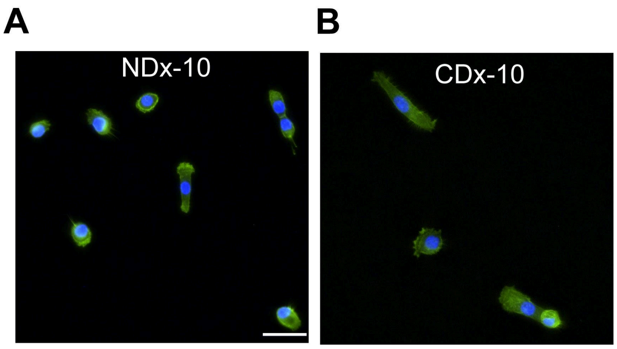

Figure S16. Images of cells in one field of view for MCF10A mammary epithelial cells. (A) NDx-10 (B) CDx-10. Scale bar represents 50 μm.

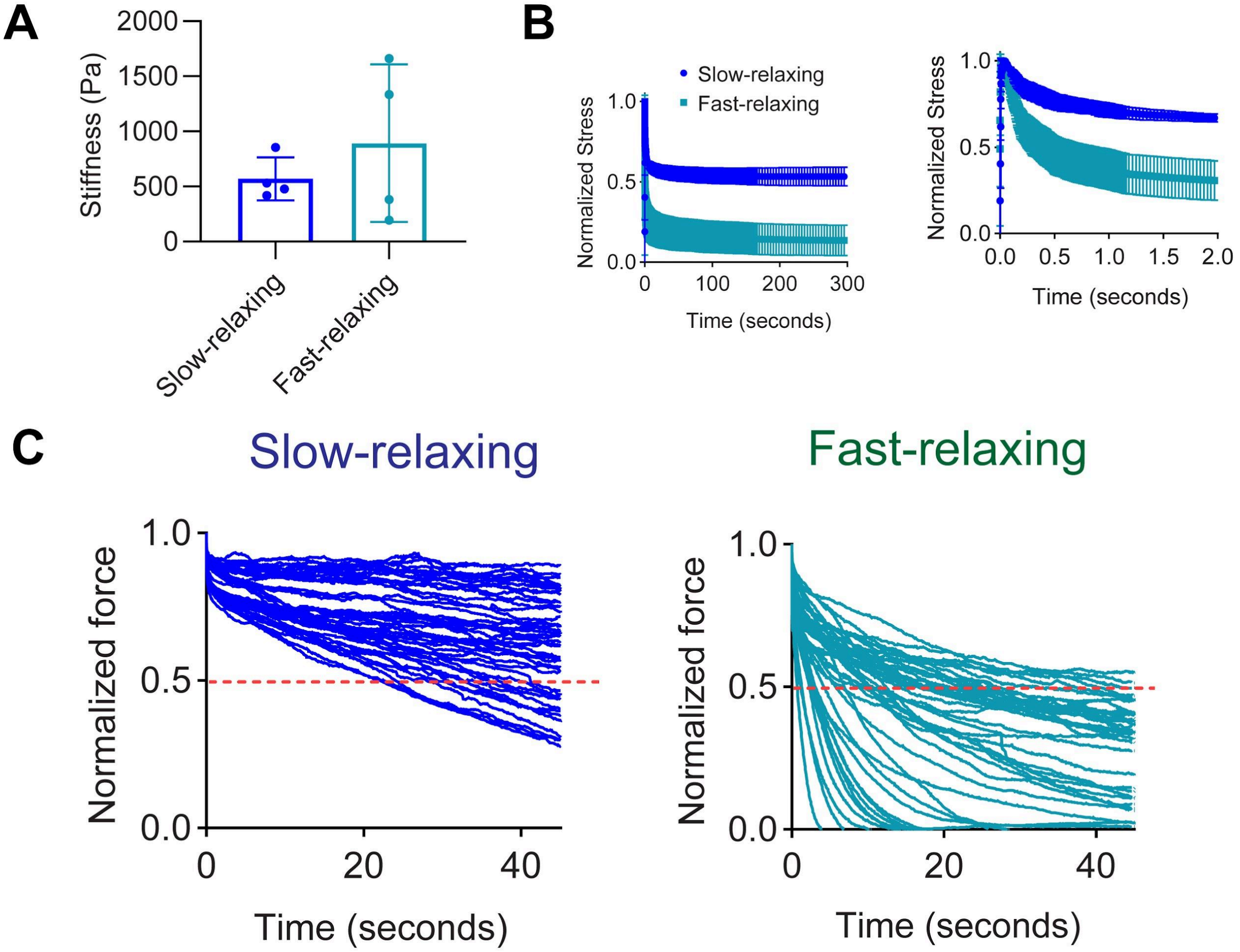

Figure S17. Mechanical characterization of poly(acrylamide) gels. (A) Stiffness of gels at 0.1 Hz characterized using frequency sweep rheology. (B) Stress-relaxation of gels characterized by using a strain of 5% using rheology. (C) AFM raw relaxation curves characterized by applying a force of 2 nN followed by monitoring relaxation for 50 seconds.
